# scMC learns biological variation through the alignment of multiple single-cell genomics datasets

**DOI:** 10.1186/s13059-020-02238-2

**Published:** 2021-01-04

**Authors:** Lihua Zhang, Qing Nie

**Affiliations:** 1 Department of Mathematics, University of California, Irvine, CA 92697 USA; 2 NSF-Simons Center for Multiscale Cell Fate Research, University of California, Irvine, CA 92697 USA; 3 Department of Developmental and Cell Biology, University of California, Irvine, CA 92697 USA

**Keywords:** Single-cell genomics data, Data integration, Biological variation, Technical variation, Batch effect removal

## Abstract

Distinguishing biological from technical variation is crucial when integrating and comparing single-cell genomics datasets across different experiments. Existing methods lack the capability in explicitly distinguishing these two variations, often leading to the removal of both variations. Here, we present an integration method scMC to remove the technical variation while preserving the intrinsic biological variation. scMC learns biological variation via variance analysis to subtract technical variation inferred in an unsupervised manner. Application of scMC to both simulated and real datasets from single-cell RNA-seq and ATAC-seq experiments demonstrates its capability of detecting context-shared and context-specific biological signals via accurate alignment.

## Background

The number and the variety of single-cell genomics datasets have grown tremendously in recent years [1]. Due to rapid technological development, single-cell datasets have been generated under different experiments and sometimes produced by different technological platforms. Particularly, an increasing number of single-cell datasets have been collected under different biological conditions, e.g., control vs. perturbation. Direct comparisons of those datasets are important for better understanding how distinct cell states respond to perturbation or disease. However, such integrative comparison analysis remains challenging due to the inevitable mixture of technical variation (or batch effects) and biological variation [2–4].

Different batch effect correction or data integration methods have been recently developed for single-cell RNA sequencing (scRNA-seq) data [5–16]. For example, mnnCorrect removes batch effects using the Gaussian smoothing of batch vectors, which are estimated by computing mutual nearest neighbors (MNNs) [6]. LIGER infers shared and dataset-specific factors based on an integrative non-negative matrix factorization method and performs alignment using the shared factors after quantile normalization [8]. Seurat V3 uses canonical correlation analysis (CCA) to identify shared correlation structures across different datasets [9]. CCA maximizes the correlation between different datasets after projection, and the aligned basis vectors are applied to all datasets to mitigate both technical and biological differences. Harmony groups cells into multi-dataset clusters and iteratively learns cell-specific linear correction factors, producing a shared embedding in which batch effects are removed [10]. A benchmarking study showed that LIGER, Seurat V3, and Harmony perform better than other existing methods through comprehensive comparisons among 14 state-of-the-art methods [17]. However, recent large-scale benchmarking studies [18, 19] suggested that method performance is dependent on the complexity of the integration task and multiple batches introduce additional difficulties for those methods that perform well for two batches. Moreover, these methods did not explicitly distinguish technical variation from biological variation when aligning multiple single-cell datasets, which might mitigate biological variation as well when removing technical variation. In such case, these methods might favor the removal of batch effects over conservation of biological variation, leading to failure in detecting cell populations that only exist in one biological condition.

In this study, we introduce a novel single-cell data integration method to match and compare (scMC) multiple single-cell genomics datasets. scMC first detects putative cell clusters for each dataset separately, then deconvolutes technical and biological variation for each pair of cell clusters across any two datasets, and finally learns correction vectors by using variance analysis to subtract the inferred technical variation. The corrected data outputted from scMC is a shared reduced dimensional embedding of cells that retains biological variation while removing technical variation. This corrected data facilitates other major tasks of single-cell analysis such as low-dimensional visualization, cell clustering, and pseudotemporal trajectory inference. We compare scMC with three data integration methods, including LIGER, Seurat V3, and Harmony, on both simulated and real datasets from single-cell RNA-seq and ATAC-seq experiments. We demonstrate the superior performance of scMC, in particular in detecting context-specific cellular heterogeneity. scMC is freely available as an R package at the GitHub repository (https://github.com/amsszlh/scMC).

## Results

### Overview of scMC

**Fig. 1:**
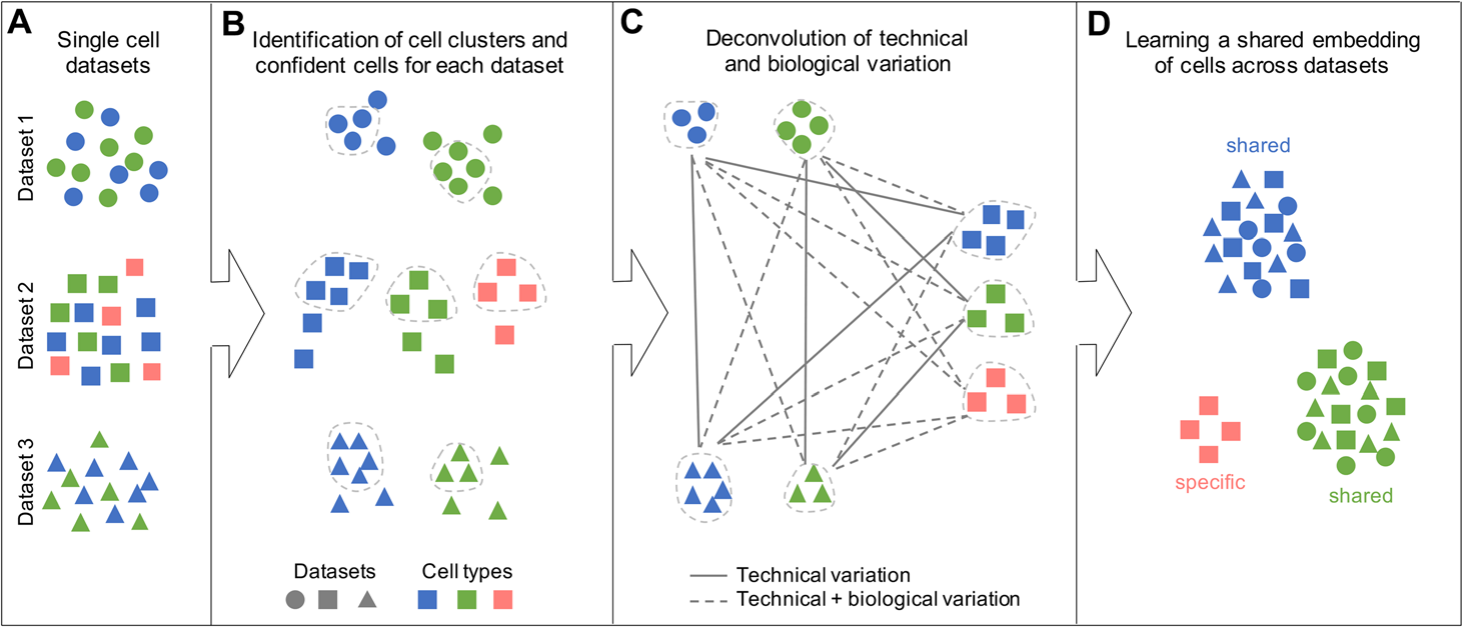
Overview of scMC. **a** scMC takes multiple single-cell genomics datasets as input. Datasets and cell types are represented by different shapes and colors, respectively. **b** scMC identifies putative cell clusters for each dataset using a Leiden algorithm-based consensus approach and defines a set of confident cells in each cell cluster, as indicated by the cells inside the dashed lines. **c** scMC deconvolutes technical variation by identifying all pairs of shared cell clusters across any two datasets based on their similarity. The differences between any other pairs of cell clusters are attributed to both technical and biological variation, as indicated by the dashed lines. **d** scMC learns a shared embedding of cells by subtracting the inferred technical variation using variance analysis. In this shared embedding space, cells are grouped by cell types rather than dataset batches, allowing detection of dataset-shared and dataset-specific biological signals

scMC takes as input multiple single-cell datasets, which may be single-cell transcriptomic or epigenomic data from different conditions, individuals, or time points (Fig. 1a). scMC first respectively groups cells into putative clusters for each dataset using a Leiden community detection method [20] (Fig. 1b). A consensus strategy is developed to assign cells into clusters, to account for the different resolution parameters in the Leiden algorithm (“Methods”). Second, scMC detects confident cells that exhibit high similarity with other cells in each cell cluster (Fig. 1b). The cell-cell similarity is quantified by the fraction of shared nearest neighbors between a cell and its neighbors, and the average similarity associated with each cell is computed (“Methods”). Confident cells are defined as the set of cells with its average similarity greater than the third quantile of average similarities of all cells within each cluster. Third, scMC infers shared cell clusters between any pair of datasets by learning a weighted complete bipartite graph connecting different cell clusters. The edge weight of the graph represents the similarity between two cell clusters, which is dependent on the similarity of the transcriptomic or epigenomic profiles and the shared features for the identified confident cells (“Methods”). A pair of shared cell clusters is then identified if they have high similarity (“Methods”). Any differences between these shared cell clusters are considered as technical variation (i.e., batch effects), while differences between any other pairs of cell clusters are attributed to both technical and biological variation (Fig. 1c). Fourth, scMC unveils a set of correction vectors by explicitly subtracting the technical variation using variance analysis for the identified confident cells (“Methods”). Finally, a corrected data is obtained by projecting the original data onto the learned correction vectors, in which the biological variation is retained while removing the technical variation (Fig. 1d). We emphasize that this correction is performed for all cells rather than confident cells only. This corrected data is a shared embedding of cells from multiple datasets and can be used for various downstream analysis such as low-dimensional visualization, cell cluster identification and pseudotemporal trajectory inference.

### scMC outperforms other state-of-the-art methods under different simulation scenarios

We first benchmark scMC against three other state-of-the-art integration methods, including LIGER, Seurat V3, and Harmony, by simulating six single-cell datasets using Splatter package [21]. Detailed descriptions of the simulated datasets are shown in Additional file 1. Each dataset is one of the six scenarios with different properties: dataset 1 contains two batches with the same cell subpopulation compositions; dataset 2 contains two batches with imbalanced cell subpopulation compositions; dataset 3 contains three batches with imbalanced cell subpopulation compositions; dataset 4 contains two batches and describes a continuous cell trajectory; dataset 5 and dataset 6 are large-scale datasets with tens of thousands of cells across multiple batches: dataset 5 contains six batches and dataset 6 has high batch complexity with 16 nested sub-batches (four sets of four sub-batches).

Using 16 evaluation metrics, we evaluated these four methods in terms of their ability to remove batch effects while maintaining cell subpopulation separation. Similar to a recent benchmarking study [19], these 16 metrics were divided into two categories assessing batch effect removal and biological variation conservation respectively (“Methods”). Batch effect removal was assessed by 5 metrics, including PCR batch, batch ASW, graph iLISI, kBET, and graph connectivity [19]. Biological variation conservation, which can be captured based on the cell identity labels, was measured via 10 metrics, including label ASW, isolated label F1, isolated label silhouette, graph cLISI, NMI label, ARI label, Jaccard label, Purity label, silhouette score, and specificity score [19] (“Methods”). A LISI-derived F1 score was defined to assess both batch effect removal and biological variation conservation (“Methods”). Moreover, we defined an overall accuracy score by averaging the rank of each integration method based on three categories of metrics, including batch effect removal metrics, biological conservation metrics, and both batch effect removal and biological conservation metrics. A larger overall score indicates better performance (“Methods”).

**Fig. 2:**
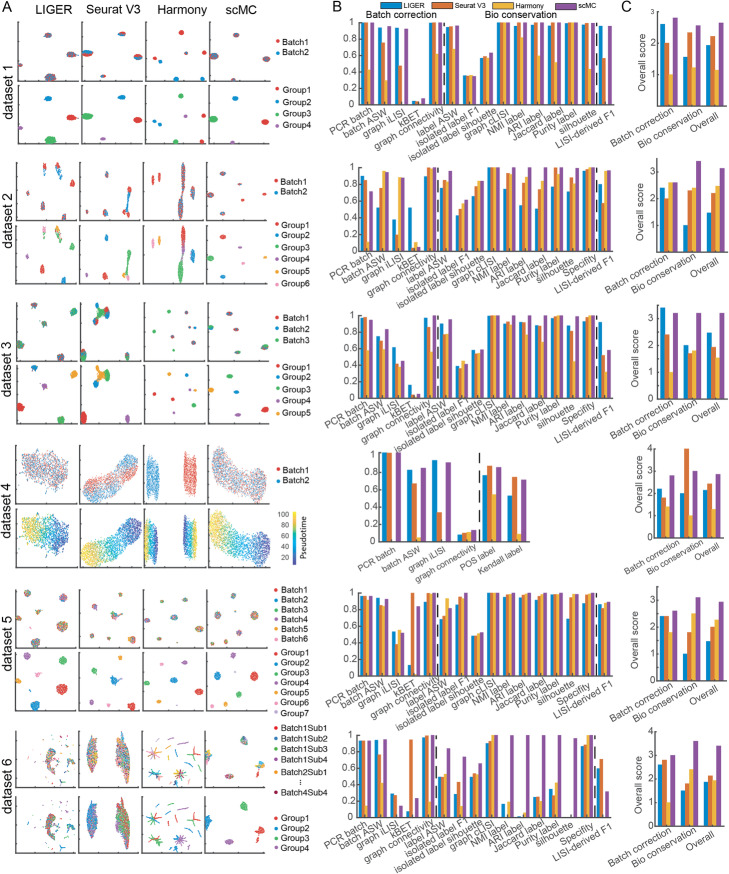
Comparisons of scMC against other methods on six simulation scenarios. **a** UMAP visualization of the corrected data from LIGER, Seurat V3, Harmony, and scMC. For each dataset, cells are colored by batch labels (top row) and gold standard cell labels or pseudotime (bottom row). **b** Evaluation of integration methods using 16 metrics in two categories for measuring batch effect removal (i.e., Batch correction) and biological variation conservation (i.e., Bio conservation). LISI-derived F1 score is a summarized metric assessing both batch mixing and cell type separation. The consistency between the inferred pseudotime from the corrected data and the gold standard pseudotime is computed using two metrics POS and Kendall rank correlation coefficient. **c** Comparison of the overall scores among different methods calculated based on batch effect removal metrics, biological variation conservation metrics, and both batch effect removal and biological variation conservation metrics

On dataset 1 with the same number of cell subpopulations across two batches, both Seurat V3 and scMC successfully removed batch effects and separated distinct cell subpopulations, as shown in the Uniform Manifold Approximation and Projection (UMAP) [22] space and reflected in their relatively high batch removal and biological conservation scores (the first row of Fig. 2a–c). However, LIGER showed a mixture of cells between different cell subpopulations, leading to lower biological conservation scores and Harmony failed to merge different batches, leading to lower batch effect removal scores. As expected, scMC showed consistently higher biological conservation scores as well as the highest overall scores in both batch effect removal and biological conservation (the first row of Fig. 2b, c). On dataset 2 with imbalanced cell subpopulation compositions across two batches, i.e. six subpopulations in batch 1 and four subpopulations in batch 2, LIGER and Seurat V3 removed batch effects at the expense of a strong loss of biological variation, where they incorrectly aligned the imbalanced cell subpopulations (the second row of Fig. 2a), leading to consistently lower biological conservation scores, in particular for the isolated label F1 and Specificity scores (the second row of Fig. 2b). In contrast, Harmony and scMC showed a good compromise between removal of batch effects and conservation of biological variation, as reflected in their moderate batch removal and higher biological conservation scores. Of note, Harmony changed the data structure and made the various cell subpopulations more compact with some mixed cell labels, leading to lower label ASW, NMI, ARI, Jaccard, and Purity scores (the second row of Fig. 2b). Together, scMC has comparable performance with some existing methods on the balanced datasets; however, it exhibits superior performance on the imbalanced datasets.

scMC integrates multiple single-cell datasets independent of the order of input datasets. On dataset 3 with three batches and imbalanced cell subpopulations, i.e., four subpopulations in batch 1, five subpopulations in batch 2, and three subpopulations in batch 3, both Seurat V3 and Harmony partially merged cells from different batches, whereas LIGER and scMC succeeded in removing batch effects from all these three batches, leading to higher batch removal scores (the third row of Fig. 2a–c). When assessing the conservation of cell subpopulations, LIGER showed a mixture of cell labels, leading to lower subpopulation conservation scores compared to scMC.

We also evaluated the conservation of cell trajectory using dataset 4 with two batches. In comparison to Seurat V3 and Harmony that either partially merged or completely retained the two batches, LIGER and scMC successfully removed the batch effects, leading to higher batch removal scores (the fourth row of Fig. 2a–c). Nevertheless, LIGER lost the trajectory structure and did not correctly reflect the pseudotemporal order of cells (the fourth row of Fig. 2a). This observation was further confirmed when we computed the consistency between the pseudotime inferred from the corrected data and the gold standard pseudotime, which was evaluated by two metrics: *POS* and Kendall rank correlation coefficient (“Methods”, the fourth row of Fig. 2b).

To evaluate the ability of dealing with multiple batches in large-scale datasets, we tested these methods on simulation datasets 5 and 6. By visually scrutinizing the projected cells in the integrated UMAP space, on simulation dataset 5, all integration methods including LIGER, Seurat V3, Harmony, and scMC could remove batch effects and detect real cell subpopulations. However, scMC exhibits better performance in preserving cell identity labels than other methods. LIGER, Seurat V3, and Harmony clearly misplaced some cells in the integrated UMAP space (the fifth row of Fig. 2a). Quantitatively, comparing to LIGER, Seurat V3, and Harmony, scMC consistently showed higher biological conservation scores using various metrics (the fifth row of Fig. 2b) and had the highest overall scores in both batch effect removal and biological conservation (the fifth row of Fig. 2c). On simulation dataset 6 with complex batch effects including 16 nested sub-batches, LIGER and Seurat V3 removed batch effects but showed a complete mixture of cell subpopulations (the sixth row of Fig. 2a), leading to higher batch removal scores and lower biological conservation scores (the sixth row of Fig. 2b, c). Harmony partially merged different batches and retained biological variation with some mixed cell labels (the sixth row of Fig. 2a). Nevertheless, scMC could retain cell identify labels while removing batch effects, leading to moderate batch removal scores and consistently higher biological conservation scores. Based on the overall scores, scMC shows the highest scores in both batch effect removal and biological conservations (the sixth row of Fig. 2c). Together, scMC exhibits superior performance in removing batch effects while conserving biological variation at the cell subpopulation and trajectory level. Such performance is particularly noticeable for the batches with imbalanced cell subpopulation compositions.

To evaluate the computational cost of scMC, we created five simulation datasets with 1000, 5000, 10,000, 20,000, and 30,000 cells using Splatter package [21] (Additional file 1). Among the compared methods, Harmony and Seurat V3 need less runtime compared to other two algorithms. The runtimes for scMC and LIGER are comparable (Additional file 2: Figure S1). For example, for a dataset with 10,000 cells, the runtimes of LIGER, Seurat V3, Harmony, and scMC are 26, 4.63, 1.72, and 9.9 min, respectively.

### scMC robustly predicts PBMC subpopulation perturbation across conditions

To assess the ability of scMC on aligning cell subpopulations across different biological conditions on real datasets, we integrated scRNA-seq data from 13,999 human peripheral blood mononuclear cells (PBMCs) for both control and interferon *β*-stimulated conditions (denoted by control condition and stimulated condition hereafter) [23]. Previous studies revealed the same cell subpopulation compositions across the two conditions, including 13 subpopulations in both conditions [24]. For this case, scMC had comparable performance with LIGER, Seurat V3, and Harmony in removing batch effects and preserving biological variation (Additional file 2: Figure S2). Besides evaluating the integration performance using all the cells in both conditions, we also made comparisons on 26 in silico datasets derived directly from PBMC datasets, which simulate vast differences in cell subpopulation compositions across conditions.

**Fig. 3:**
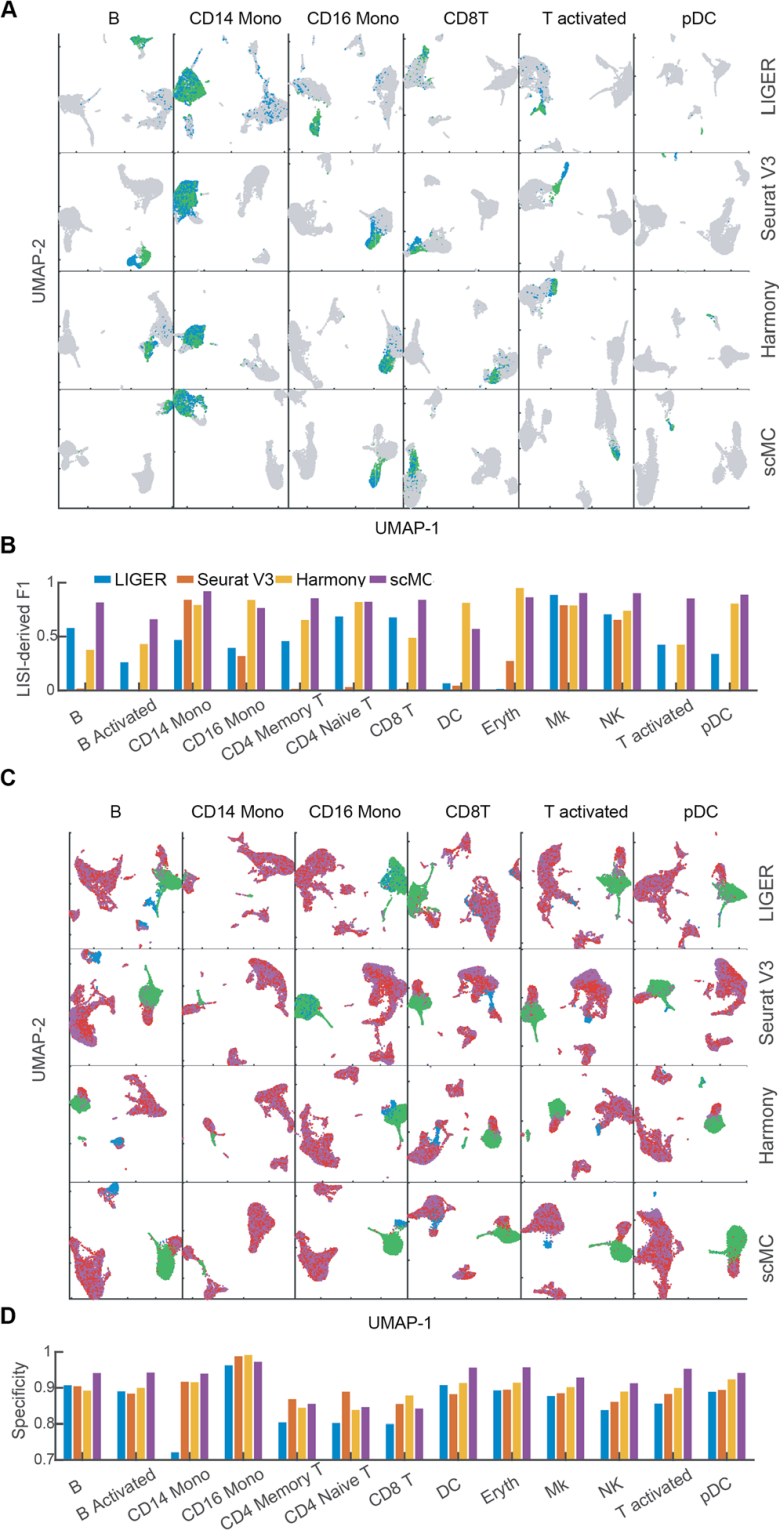
scMC aligns and preserves condition-specific cell subpopulations on perturbed PBMC datasets. **a** UMAP of the corrected data from LIGER, Seurat V3, Harmony, and scMC across control and stimulated conditions in the perturbed PBMC datasets. Each row represents one method, and each column represents one perturbed dataset in which only one cell subpopulation was retained in the control condition (indicated on the top). See Additional file 2: Figure S3A for other 7 perturbed datasets. Green: cells retained in the control condition; blue: cells from the corresponding same cell subpopulation in the stimulated condition; gray: other cells in the stimulated condition. **b** LISI-derived F1 scores of LIGER, Seurat V3, Harmony, and scMC on all 13 perturbed datasets. **c** UMAP of the corrected data from LIGER, Seurat V3, Harmony, and scMC across control and stimulated conditions. Each column represents one perturbed dataset, where the cell subpopulation removed in the control condition is labeled on the top, and CD14 Mono and DC cell subpopulations were also removed in the stimulated condition for all cases. See Additional file 2: Figure S3B for other 7 perturbed datasets. Green: CD14 Mono and DC cells from the control condition. Red: other cells from the control condition. Blue: cells of the cell subpopulation removed from the control condition in the stimulated condition. Purple: other cells in the stimulated condition. **d** Specificity scores of LIGER, Seurat V3, Harmony, and scMC on all 13 perturbed datasets

To assess the ability of alignment in merging few shared cell subpopulations across conditions, we first created 13 datasets by retaining one of the 13 subpopulations in control (e.g., removal of all cells except for DC cells from control condition) but keeping all cell subpopulations in stimulated condition. Ideally, a good alignment method should merge the cells from control condition with the cells of the same cell subpopulation from stimulated condition. By visualizing the aligned data in the UMAP space, we found that scMC, compared to LIGER, Seurat V3, and Harmony, better aligned the cell subpopulation from control condition (green) and the corresponding cell subpopulation from stimulated condition (blue) on most of the perturbed datasets (Fig. 3a, Additional file 2: Figure S3A). Moreover, we used LISI-derived F1 score to quantitatively assess both batch mixing and cell type separation (“Methods”). scMC had a higher LISI-derived F1 score than other methods on 10 out of 13 perturbed datasets (Fig. 3b, Additional file 2: Figure S4). These results demonstrate the scMC’s ability in removing batch effects and retaining biological variation for the datasets with only few shared cell types.

To assess the ability of alignment in preserving subtle biological differences across conditions, we created another 13 perturbed datasets by removing one cell subpopulation from control condition and two cell subpopulations from stimulated condition, to ensure each condition with at least one specific cell subpopulation in all perturbed datasets. For the stimulated condition, we removed CD14 Mono and DC cell subpopulations, representing larger (31%) and smaller (3.6%) cell populations. By visualizing the aligned data in the UMAP space, we found that only scMC robustly separated the condition-specific cell subpopulations from other cells. Nevertheless, LIGER merged the stimulated-specific cell subpopulation with other cells from both conditions, and Seurat V3 and Harmony merged the control-specific cell subpopulations (i.e., either CD14 Mono or DC) with other cells from stimulated condition, on most of the perturbed datasets (Fig. 3c, Additional file 2: Figure S3B). Additionally, we quantitatively evaluated the ability of preserving condition-specific cell types by defining a specificity score, which assesses the overlap ratio between the cells within condition-specific cell types with other cells based on their nearest neighbors in a low-dimensional space (“Methods”). scMC had higher specificity scores than other methods on 9 out of 13 perturbed datasets (Fig. 3d). Together, scMC exhibits better performance in preserving condition-specific cell types than other compared methods, which often favor the removal of batch effects over conservation of biological variation.

### scMC predicts a specific fibroblast subpopulation upon Hedgehog activation during mouse skin wound healing

**Fig. 4:**
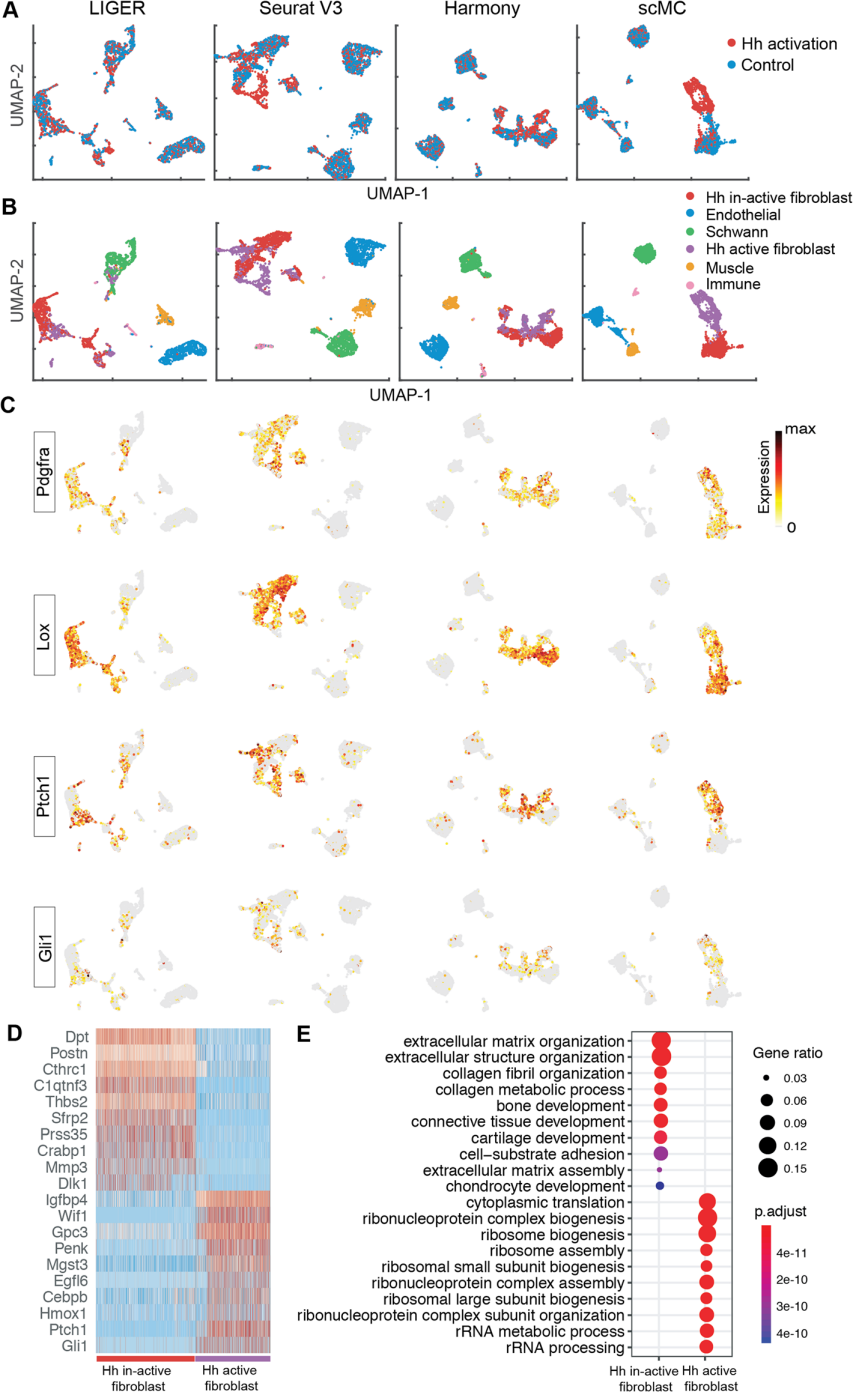
scMC reveals specific fibroblast subpopulations in control and Hedgehog activation during mouse skin wound healing. **a**, **b** UMAP of the corrected data from LIGER, Seurat V3, Harmony, and scMC across two conditions. **a** Cells are colored by experimental conditions. **b** Cells are colored based on the annotated cell labels determined based on the cell clusters identified by scMC by examining the expression patterns of known markers (Additional file 2: Figure S5). **c** Overlay the expression levels of fibroblasts pan-markers (Pdgfra and Lox) and Hh-active fibroblast markers (Ptch1 and Gil1) onto the UMAP space of LIGER, Seurat V3, Harmony, and scMC, respectively. Dark red and gray colors represent the high and zero expression, respectively. **d** Heatmap showing the expression patterns of the top 10 marker genes enriched in Hh-inactive and Hh-active fibroblast subpopulations. **e** The top 10 enriched GO biological processes of the marker genes associated with the Hh-inactive and Hh-active fibroblast subpopulations

We further assessed the ability of scMC in detecting condition-specific cell subpopulations using a mouse skin scRNA-seq dataset. This dataset included cells from control and Hedgehog (Hh) activation conditions during skin wound healing [25]. Although LIGER strongly removed the batch effects across conditions (Fig. 4a), it was unable to preserve the biologically meaningful subpopulations (Fig. 4b). For example, the fibroblast cells, marked by high expression of Pdgfra and Lox, were mixed with the Schwann cells in the UMAP space, not showing cell-type-specific localization (Fig. 4b, c). In contrast, Seurat V3, Harmony, and scMC were able to separate broad cell types, including Immune, Schwann, Muscle, Fibroblast, and Endothelial cells, marked by the high expression of Cd68, Plp1, Myh11, Pdgfra, and Pecam1 (Fig. 4b, c, Additional file 2: Figure S5). Fibroblast cells from control and Hh activation conditions were not completely mixed and showed condition-specific localization in the UMAP space, suggesting the existence of condition-specific fibroblast subpopulations (Fig. 4b). Intriguingly, scMC was able to differentiate the Hh-active from Hh-inactive fibroblast populations, in which the Hh-active fibroblasts were enriched for markers such as Ptch1 and Gil1, whereas other methods failed to do so with a number of mixed cells (Fig. 4b, c). Together, through integration across conditions, scMC detects a specific fibroblast subpopulation upon Hh activation during mouse skin wound healing.

To investigate biological differences between the new identified Hh-active fibroblast subpopulation and the Hh-inactive fibroblast subpopulation (i.e., the classic fibroblasts), we performed differential expression analysis between these two subpopulations as well as functional enrichment analysis of the marker genes associated with each subpopulation using ClusterProfiler R package [26]. These two fibroblast subpopulations are not only enriched for very different signature genes (Fig. 4d) but also vast different biological processes (Fig. 4e). The Hh-inactive fibroblasts are involved in typical processes such as extracellular matrix (ECM) and collagen formation associated with effective wound healing. Nevertheless, Hh-active fibroblasts are involved in processes such as ribosome biogenesis/assembly and rRNA processing (Fig. 4e), implying active RNA dynamics and cell fate transition. Indeed, Hh-active fibroblasts also express dermal papilla signature genes such as Igfbp4 and Wif1, suggesting that these fibroblasts are likely undergoing transition to dermal papilla. This is in agreement with the observation that dermal fibroblasts could give rise to dermal papilla cells during hair follicle formation [27, 28]. These results suggest the potential role of Hh activation in inducing fibroblasts into dermal papilla cells. Together, we demonstrated scMC’s capability in preserving biological variation and revealing biologically meaningful insights through accurate integration.

### scMC reveals integrated developmental trajectories through simultaneous integration across replicates and time points

We next evaluated scMC’s ability to integrate time-course datasets with continuous trajectories, rather than discrete cell types. We analyzed 25,719 cells from mouse skin embryonic development at embryonic day E13.5 and E14.5, where each time point consisted of two biological replicates [29]. We performed two integration tasks: integrating the replicate samples of each time point individually and integrating all the four samples at once. First, for each time point, we integrated the scRNA-seq datasets across replicates. After integration by LIGER, Seurat V3, Harmony, and scMC, all these methods removed the batch effects between the two replicates and revealed major cell populations in embryonic skin, such as epidermal and dermal populations (Additional file 2: Figure S6-S7).

**Fig. 5:**
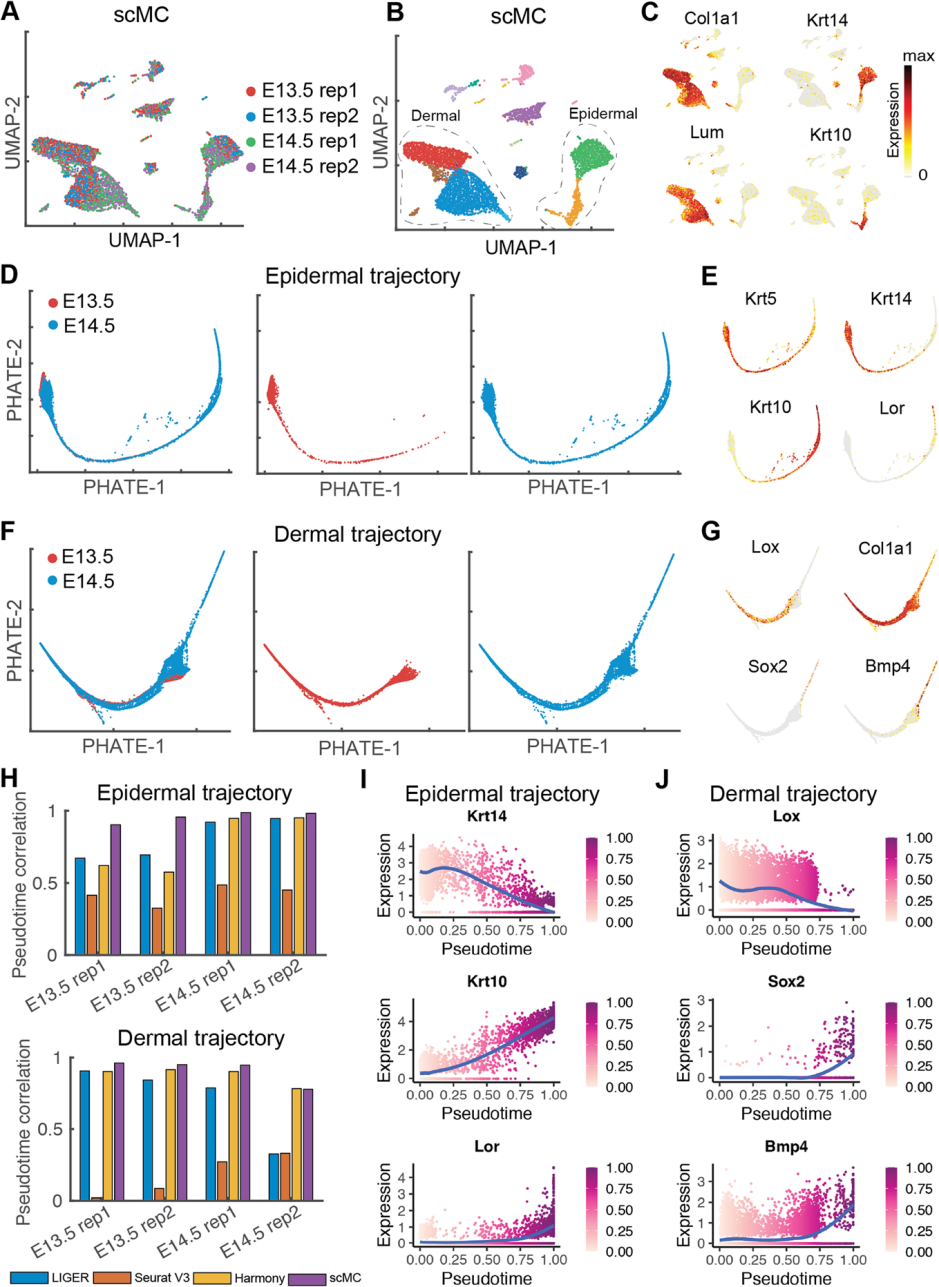
scMC reveals integrated epidermal and dermal trajectories by simultaneous integration across replicates and time points during skin embryonic development. **a**, **b** UMAP of the corrected data from scMC on the time-course scRNA-seq datasets from E13.5 to E14.5. **a** Cells are colored by the replicates and time points. **b** Cells are colored by the identified cell subpopulations from the corrected data. Cells inside the dashed line were identified as dermal and epidermal cells based on their known markers. **c** Overlay the expression levels of markers of dermal (Col1a1 and Lum) and epidermal cells (Krt14 and Krt10) onto the UMAP space. Dark red and gray colors represent the high and zero expression, respectively. **d** PHATE visualizations for the epidermal cells from both E13.5 and E14.5, only E13.5, and only E14.5, respectively. **e** Overlay the expression levels of markers of epidermal cells (Krt5, Krt14, Krt10, and Lor) onto the PHATE space. **f** PHATE visualizations for the dermal cells from both E13.5 and E14.5, only E13.5, and only E14.5, respectively. **g** Overlay the expression levels of markers of dermal cells (Lox and Col1a1) and DC cells (Sox2 and Bmp4) onto the PHATE space. **h** Comparison of the recovered trajectories by computing Pearson correlation coefficients between the pseudotime values of cells from each replicate sample before and after integration. **i, j** Pseudotemporal dynamics of un-differentiation and differentiation marker genes reconstructed from the integrated trajectories. Cells are colored based on the pseudotime values. Blue lines represent the locally weighted smoothing expression. Color bar represents the scaled pseudotime values

Next, we reconstructed the integrated epidermal and dermal trajectories using all the four samples, which were potentially challenging due to the need of simultaneous integration across replicates and time points. By integrating all the four samples from E13.5 and E14.5 using LIGER, Seurat V3, Harmony, and scMC, we were able to extract the epidermal and dermal cells based on their known makers such as Col1a1, Lum, Krt14, and Krt1 (Fig. 5a–c, Additional file 2: Figure S8-S10A-C). We then reconstructed the epidermal and dermal trajectories by applying a diffusion-based manifold learning method PHATE [30, 31] to the corrected data from each integration method. For the epidermal trajectory, LIGER, Harmony, and scMC were able to produce an integrated global trajectory structure starting from basal epidermal cells and progressing toward Lor-high suprabasal epidermal cells (Fig. 5d, e, Additional file 2: Figure S8 and S10D-E). However, the trajectory structure was lost when visualizing the corrected data from Seurat V3 (Additional file 2: Figure S9D-E). For the dermal trajectory, scMC placed cells in an expected differentiation trajectory, which recapitulates sequential stages of dermal cell lineage differentiation process from an undifferentiated dermal state to differentiated Sox2-high dermal condensate (DC) cells (Fig. 5f–g). Notably, this integrated trajectory showed that both E13.5 and E14.5 dermal cells were concentrated toward the origin of the trajectory, but E14.5 dermal cells exclusively made up the terminus of the trajectory, suggesting the specific cell fate at E14.5, which was consistent with prior knowledge [29]. However, other methods, including LIGER and Seurat V3, tended to strongly remove both batch effects and biological variation, leading to a loss of trajectory structure and failure of detecting specific cell states at E14.5 (Additional file 2: Figure S8-S9D-G). Harmony appeared to place the dermal cells in the correct order in the PHATE space although the detailed trajectory structure is unclear (Additional file 2: Figure S10D-G).

To compare the recovered trajectories in a quantitative fashion, we used a similar method from Luecken et al. [19], which assumed that trajectories found in the unintegrated data for each batch gave the most accurate biological signal. Thus, we computed Pearson correlation coefficients between the pseudotime values of cells in each replicate sample before and after integration (“Methods”). Consistent with our visual observation, LIGER, Harmony, and scMC performed well in preserving cell pseudotime for the epidermal trajectory, in particular for the cells from E14.5 at which cells can progress toward the suprabasal cell state (Fig. 5h). For the dermal trajectory, although Harmony did not produce a clear trajectory in the PHATE space (Additional file 2: Figure S10F), it showed comparable correlation values with scMC (Fig. 5h). However, LIGER and Seurat V3 produced lower correlation values for the dermal trajectory due to the loss of trajectory structure (Fig. 5h).

In addition, we investigated the pseudotemporal dynamics of several marker genes based on the scMC-derived integrated developmental trajectories. As expected, during epidermal and dermal differentiation, the temporal dynamics of undifferentiated (e.g., Krt14 and Lum) and differentiated (e.g., Krt10, Lor, Sox2 and Bmp4) markers were downregulated and upregulated over pseudotime, respectively (Fig. 5i, j). scMC correctly captures the developmental process using datasets from different time points and replicates, showing meaningful gene dynamics.

### scMC is able to deal with multiple batches in complex datasets

**Fig. 6:**
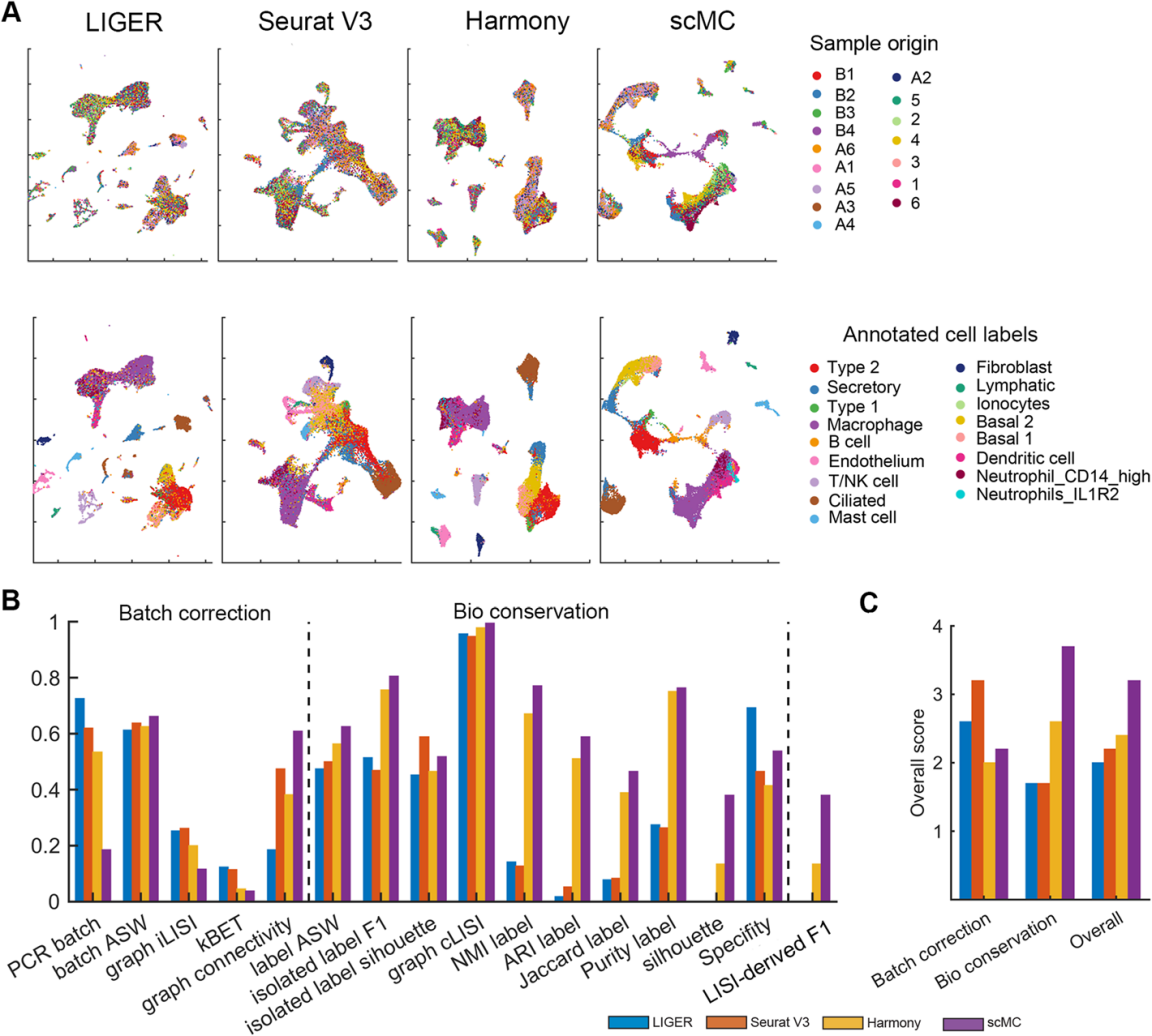
scMC is able to integrate a complex mouse lung scRNA-seq dataset across 16 batches. **a** UMAP of the corrected data from LIGER, Seurat V3, Harmony, and scMC on a mouse lung scRNA-seq dataset across 16 batches. Cells are colored by known sample origins (top panel) and annotated cell labels (bottom panel), respectively. **b** Evaluation of integration methods using 16 metrics, grouped into two categories: batch effect removal (i.e., Batch correction) and biological variation conservation (i.e., Bio conservation). LISI-derived F1 score is a summarized metric assessing both batch mixing and cell type separation. **c** Comparison of over scores among different methods

To further assess the ability of scMC in integrating complex scRNA-seq dataset with multiple batches, we applied scMC to a mouse lung scRNA-seq dataset with 32,472 cells across 16 batches, which was used in a recent benchmarking study [19]. The batch labels (i.e., sample origins) and annotated cell labels were used to compute evaluation metrics. All the tested methods performed well in batch effect removal. In particular, Seurat V3 had the highest overall batch removal score while scMC exhibited a moderate batch removal score in comparison with other methods (Fig. 6a–c). However, Seurat V3 performed poorly in preserving the biological variation, making various cell subpopulations more compact in the UMAP space along with some mixed cell labels and leading to lower biological conservation scores. Seurat V3 significantly reduced the biological variation among different cell types, such as epithelial cells (i.e., ciliated cells, basal 1 and basal 2), fibroblasts, and immune cells (i.e., B cells, endothelial cells and mast cells). Nevertheless, LIGER, Harmony, and scMC succeeded in separating these broad cell types. Compared to scMC with the highest biological conservation scores, LIGER separated the ciliated cells into different groups and was not able to discriminate basal 1 from basal 2 cells and neutrophils from macrophage, leading to the lowest biological conservation scores, in particular for ASW label and ARI label. Harmony exhibited good separation of different cell types, but showed an overlap between neutrophils, dendritic cells, and macrophage, leading to relatively lower biological conservation scores compared to scMC. scMC can not only separate different broad cell types, but also discriminate biologically similar cell states, such as basal 1 and basal 2, macrophage, and dendritic cells. Quantitatively, scMC showed moderate batch removal scores, but also the highest biological conservation scores as well as the overall score (Fig. 6c).

### scMC consistently performs well in integrating single-cell ATAC-seq data

We next assessed whether the capability and performance of scMC can transfer to other modalities, such as single-cell ATAC-seq data. Additional challenge here is the extremely sparse and near-binary characteristics in scATAC-seq data, for which we first transformed the chromatin accessibility data into other informative feature matrix, including *k*-mers and genes (“Methods”). These two feature matrices were constructed using *k*-mer-based chromVAR [32] and Gene Scoring [33]. We gathered single-cell chromatin profiles from four adult mouse tissues (whole brain, large intestine, heart, and liver), in which the whole brain and large intestine had two replicates [34]. A good alignment method should merge cells from different replicates in the same tissue as well as preserve biological variation among different tissues. To evaluate the ability of preserving condition-specific cells, we treated cells from the whole brain replicate 1, large intestine replicate 1, heart, and liver as condition 1, and cells from the whole brain replicate 2 and large intestine replicate 2 as condition 2.

**Fig. 7:**
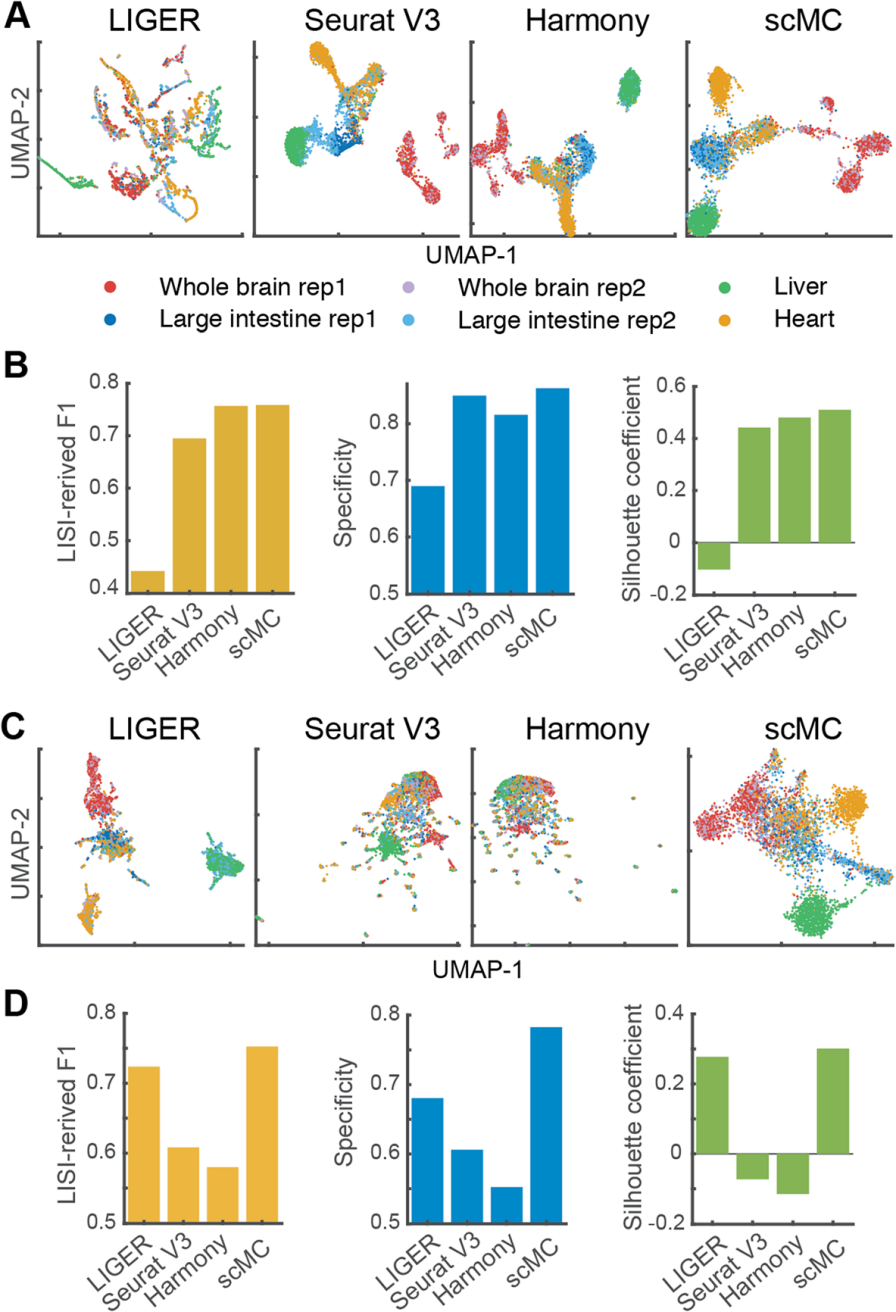
scMC performs well in integrating scATAC-seq datasets. **a** UMAP of the corrected data from LIGER, Seurat V3, Harmony, and scMC on an scATAC-seq dataset with the feature matrix transformed by ChromVAR. Cells are colored by known tissue origins. **b** Quantitative evaluation of removing batch effects, preserving condition-specific tissues, and separating different tissues on the aligned UMAP space from four methods using LISI-derived F1, specificity, and silhouette metrics. The feature matrix was constructed using ChromVAR. **c**, **d** The four integration methods applied to the feature matrix transformed by Gene Scoring

First, we applied the integration methods to the chromVAR-transformed feature matrix (“Methods”) and analyzed the corrected data in the UMAP space. scMC removed the batch effects between different replicates from the same tissue while retaining the biological differences among different tissues (Fig. 7a, Additional file 2: Figure S11A), leading to the higher batch effect removal and biological conservation scores (Additional file 2: Figure S12A-B). Both LIGER and Harmony merged the liver and heart cells with the whole brain and large intestine cells, suggesting that biological variation was also removed along with the batch effects. Seurat V3 neither clearly distinguished liver cells from large intestine cells nor merged the two replicates from large intestine tissue (Fig. 7a), leading to the lower batch effect removal and biological conservation scores compared to scMC (Additional file 2: Figure S12A-B). Moreover, we used LISI-derived F1 metric to assess replicate mixing as well as tissue separation, and specificity metric to evaluate the ability of preserving condition-specific tissues (“Methods”). Harmony and scMC had the higher LISI-derived F1 scores than LIGER and Seurat V3 (Fig. 7b, Additional file 2: Figure S12C), suggesting better performance of Harmony and scMC in removing batch effects. However, Harmony had lower specificity score than scMC, indicating better performance of scMC in preserving condition-specific tissues than Harmony (Fig. 7b). Additionally, we assessed tissues separability in the UMAP space using the silhouette score (“Methods”) to quantify how well each method groups and separates the cells from other groups of cells. scMC showed the largest silhouette score compared to other three methods (Fig. 7b).

scMC identified four cell subpopulations in the brain tissue (Additional file 2: Figure S13A). To investigate the biological roles of these cell subpopulations, we aggregated chromatin accessible signals across each cell subpopulation and identified differential loci of these four cell subpopulations (Additional file 1). By performing gene ontology enrichment analysis using GREAT [35] and motif enrichment analysis by chromVAR [32], we found different enriched biological processes and motif patterns across these four different cell subpopulations (Additional file 2: Figure S13B-C). For example, Subcluster 1 was enriched for ectoderm development and cell differentiation, Subcluster 2 was enriched for defense response, and Subcluster 3 was enriched for T cell differentiation (Additional file 2: Figure S13C).

Second, we applied the integration methods to the Gene Scoring-transformed feature matrix (“Methods”) and found that scMC merged cells from the same tissues, distinguished condition-specific tissues, and separated different tissues in the UMAP space (Fig. 7c). LIGER was also able to separate different tissues, leading to higher biological conservation scores (Additional file 2: Figure S12D-F). Seurat V3 and Harmony completely merged cells from all tissues together (Fig. 7c), producing lower biological conservation scores (Additional file 2: Figure S12D-F). Moreover, except for scMC, other three methods completely mixed the cells from the two conditions (Additional file 2: Figure S11B). Together, scMC consistently exhibits superior performance in removing batch effects and preserving biological variation when integrating scATAC-seq datasets.

## Discussion

A rapid increase in single-cell genomics datasets collected from different conditions, experiments, and labs presents a major challenge in comparing datasets due to batch effects [6, 19]. The inevitable mixture of batch effects and biological variation, which is inherent to biological systems, introduces further challenges for comparing among different single-cell genomics datasets. For example, the three state-of-the-art integration methods LIGER, Seurat V3, and Harmony are found to often remove or reduce biological variation due to their strong batch removal capability, as shown on both simulation and real datasets. This is particularly noticeable for the batches with imbalanced cell subpopulation compositions.

Unlike existing methods, scMC can explicitly differentiate batch variation from biological variation, by inferring the batch variation in an unsupervised manner and learning the biological variation by subtracting the inferred technical variation from the total variation. In contrast, other methods, such as Seurat V3, learn a set of shared correction vectors by considering all available cells in the datasets, potentially lacking the ability in discriminating technical variation from biological variation. Together, scMC provides a reliable way to identify true biological differences from multiple datasets.

Reconstruction of integrated pseudotemporal trajectories is important yet challenging for joint analysis of developmental dynamics across datasets. Comparisons of pseudotemporal trajectories across diverse conditions measured by different datasets require specialized methods due to batch effects [36]. Dynamic time warping has been used to align two pseudotemporal trajectories: pseudotemporal trajectories of each dataset are inferred separately and then aligned to reveal timing differences across conditions [37, 38]. Such alignment might fail to detect signals that are weak in each dataset and might be sensitive to the inaccurate inference of individually inferred pseudotemporal trajectories. Reconstructing integrated pseudotemporal trajectories in a shared embedding space across datasets could really improve such temporal analysis. As shown in the mouse embryonic skin development dataset, the scMC-derived integrated trajectory showed that both E13.5 and E14.5 epidermal and dermal cells were concentrated toward the origin of the trajectory, but E14.5 cells exclusively made up the terminus of the trajectory, suggesting that the full range of epidermal and dermal differentiation program might be already established as early as E13.5. For this case, reconstructing an integrated trajectory is critical when comparing time-course datasets. However, for the time-course datasets, most integration methods including scMC do not incorporate temporal information into the inference of correction vectors. Including such temporal information will likely better align the cells in time for more faithful integrated pseudotemporal trajectories.

As seen in the above time-course datasets, our alignment method could provide biological insights that are difficult to obtain if only analyzing datasets individually. Another example is the Hedgehog-active fibroblast subpopulation identified by scMC in the mouse skin wound system. While this subpopulation could be uncovered by analyzing the datasets individually, the question on whether this subpopulation is a specific fibroblast subpopulation upon Hedgehog activation would be challenging to answer without using alignment approach. In general, many results obtained by joint analysis might be obtained by individual analysis of each dataset with additional careful effort in comparing them; however, the apparent differences seen between two individual analysis might be caused by technical variation. The capability of distinguishing technical variation from biological variation in scMC is crucial when comparing cellular compositions and trajectories across conditions and time points, which are often measured in different batches with potential technical variation.

To predict technical variation, scMC needs to detect putative cell clusters for each dataset. To enhance robustness of integration with respect to this initial clustering, two strategies have been employed. First, a consensus clustering approach is used by combining multiple clustering solutions, obtained by varying the resolution parameter values in the Leiden algorithm. Such approach allows high accuracy and robustness in clustering, similar to the SC3 method for consensus clustering of scRNA-seq data [39]. Second, a set of confident cells in each putative cell cluster are determined to filter out the cells that might be assigned into multiple clusters by different resolutions or clustering methods. Such sketching strategy not only enables robustness of the method with respect to the initial clustering but also helps to reduce computational cost.

The computing cost of scMC is dominated by solving the main optimization problem in the algorithm, which is equivalent to calculating *k* largest eigenvectors (default *k = 40*) associated with the real positive eigenvalues of a *n* × *n* matrix, where *n* is the number of all confident cells. Therefore, the computational complexity is *O* (*kn*^2^). scMC can analyze large-scale datasets, e.g., 100 K cells within 2 h. To reduce the run time, users can use fewer confident cells by adjusting the quantile cutoff parameter (e.g., from 0.75 to 0.9) or perform subsampling of single-cell datasets using a “geometric sketching” approach [40], which allows to maintain the transcriptomic heterogeneity within a dataset with a smaller subset of cells. More efficient methods and packages for calculating eigenvalues will significantly enhance the computational speed of scMC.

The correction vectors from scMC can be directly used for correcting batch effects when additional datasets become available. For the simulation dataset 2 with three batches, we run scMC on the cells from the first two batches, and then projected the cells from the third batch onto the shared UMAP space learned for the first two batches using the learned correction vectors. As expected, the cells with the same labels from the third batch are correctly placed onto this shared UMAP space (Additional file 2: Figure S14). However, projecting cells from very different sources with strong batch effects into a shared low-dimensional space may require a more advanced transfer learning approach, such as scArches [41].

Using highly variable genes (HVGs) instead of all the genes can improve the performance of integration analysis and reduce the computational cost [19]. The correction learned from selected HVGs is often sufficient to project all cells in the shared space. Sometimes, correcting more or all genes is needed in order to study details of those genes in the shared space, for example, visualization of expression profiles of individual genes in the integrated UMAP space. While the corrected data are appropriate for studying gene expression profiles in the shared space, one should be careful about whether biological signals are removed during integration [2]. Our analysis indicates that some integration methods might remove the biological signals while removing technical variation, producing corrected data with a less accurate representation of the underlying biology. To obtain more genes in the corrected data, one usually needs to select more HVGs. To study this issue, we tested two more thresholds for HVG selection when analyzing simulation dataset 1 (leading to 4457 or 10,000 genes) and the mouse skin wound healing dataset (leading to 4792 or 17,912 genes). Consistent results have been found among the three thresholds for HVG selection (Additional file 2: Figure S15). In general, more efficient methods are required if one prefers to correct all genes when analyzing their expression profiles across datasets.

The interrelated biological conditions, such as different developmental stages or normal vs. disease, usually result in biological differences in cell states and their interactions [42]. With the increasing number of the single-cell transcriptomics datasets, such as the Human Cell Atlas and the Pediatric Cell Atlas, and the availability of other single-cell genomics data, such as scATAC-seq data [43–45], robust and accurate integration methods are particularly important when dissecting how distinct cell states respond to perturbations, diseases, and evolution.

## Conclusions

We presented a new method together with an R toolkit scMC for integrating multiple single-cell genomic data across conditions, time points, tissues, and studies. scMC is particularly effective to address the over-alignment issue existing in previous integration methods. The superior alignment performance of scMC is particularly noticeable for the datasets with imbalanced cell population compositions across interrelated biological conditions. The capability of scMC in preserving biological signals while removing batch effects will facilitate biological discoveries from the ever-growing number of single-cell studies.

## Methods

### scMC algorithm

scMC, designed to integrate single-cell transcriptomic or epigenomic data across distinct datasets, includes the following main steps in its algorithm: (1) data preprocessing; (2) feature matrix construction; (3) identification of putative cell clusters for each dataset separately; (4) detection of confident cells in each putative cell cluster; (5) inference of shared cell clusters between any pair of datasets; (6) learning the confounding matrix; (7) learning the correction vectors; and (8) construction of corrected data for downstream analysis. Below, we describe each of these steps in detail.

#### Data preprocessing

For each dataset, features expressed in less than 3 cells and cells with expressed features less than 200 are removed. For scRNA-seq data, the data is normalized as follows: expression of each gene is divided by the total expression in each cell, multiplied by a scale factor (10,000 by default) and log-transformed with pseudocount 1.

#### Feature matrix construction

For scRNA-seq data, the feature matrix is constructed by selecting a set of highly variable genes (HVGs), through calculating the average expression and Fano factor for each gene, binning the average expression of all genes into 20 evenly sized groups and then normalizing the Fano factor within each bin. The Fano factor, defined as the variance divided by the mean, is a measure of dispersion. Genes with normalized Fano factor value greater than 0.05 and average expression larger than 0.01 are selected. The number of HVGs that are selected as features is typically around 2000–3000.

For scATAC-seq data, the feature matrix is constructed by transforming features based on two methods, including ChromVAR [32] and Gene Scoring [33], to deal with the inherent sparsity and high dimensionality of scATAC-seq data [46]. ChromVAR estimates the dispersion of chromatin accessibility within peaks sharing the same *k*-mers. Gene Scoring constructs a peaks-by-cells count matrix, defines regions of interest as the 50 kb upstream and downstream of gene transcription start site (TSS), and assigns each gene an accessibility score by summarizing peaks near its TSS and weighting them by an exponential decay function based on their distances to the TSS. Therefore, the chromatin accessibility data matrix is transformed into either *k*-mers-by-cells or genes-by-cells matrix.

#### Identification of putative cell clusters for each dataset separately

For each dataset, cells are grouped into putative clusters using a consensus approach based on the Leiden community detection algorithm. First, for a given resolution parameter value in the Leiden algorithm, the putative cluster memberships are identified by applying Leiden algorithm [20] to a shared nearest neighbor (SNN) graph. This SNN graph is constructed by calculating the *k*-nearest neighbors (20 by default) for each cell based on the first 40 principle components after performing principle component analysis (PCA) on the feature matrix. The fraction of shared nearest neighbors between the cell and its neighbors is then used as weights of the SNN graph. Second, for a range of resolution parameter values (e.g., from 0.1 to 0.5 with an increment being 0.1), a set of cluster memberships are obtained, and then a consensus matrix is constructed to represent the probability of two cells being in the same cluster across multiple values of the resolution parameter. Finally, the putative cell clusters are identified by applying hierarchical clustering with average linkage to the computed consensus matrix. The number of putative cell clusters is determined by the number of larger singular values that capture the larger variance of the consensus matrix via singular value decomposition. Specifically, we calculate the ratio of each singular value to the sum of all the singular values, which indicates the percentage of variance each singular value contributes to. The number of singular values that have the ratio higher than 1% is used to estimate the number of the putative cell clusters. In the studied datasets, the number of clusters ranges from 3 to 20. A single cluster usually corresponds to one cell subpopulation. It is worth noting that some cell populations might be divided into multiple clusters due to the inherit heterogeneity and the existence of different cellular states.

#### Detection of confident cells in each putative cell cluster

For each putative cluster, a set of confident cells, defined as cells with high similarity with other cells in the cluster, are obtained. The similarities of a cell with other cells are quantified by the weights of the computed SNN. For each cell, we define its average similarity by computing the average of its similarities with all other cells within the same cluster. The third quantile of the average similarities associated with all cells within a cluster is denoted by *Q*_3_. Then confident cells are the set of cells with its average similarity higher than *Q*_3_ in each cluster.

#### Inference of shared cell clusters between any pair of datasets

For a pair of dataset *i* and dataset *j*, we build a complete bipartite graph, in which each vertex represents a cell cluster and edge weight represents the similarity between two cell clusters. The similarity between two cell clusters *p* and *q* is defined by the combination of the following two strategies. First, the similarity between clusters *p* and *q* is quantified based on the overlap fraction of the marker genes associated with each cell cluster. The marker genes of a cell cluster are selected if (i) the *p* values from likelihood-ratio tests [47] are less than 0.05, (ii) the log fold-changes are higher than 0.25, and (iii) the percentage of expressed cells in the cluster is higher than 25%. The likelihood-ratio tests are performed by comparing the gene expression of confident cells in a particular cell cluster with all other cell clusters. This method can simultaneously test for changes in mean expression (conditional on the gene being expressed) and in the percentage of expressed cells [47]. Of note, the marker genes are identified using confident cells in the cluster. Then the similarity between clusters *p* and *q* is computed by *s/min(n*_*1*_*, n*_*2*_*)*, where *s* is the number of marker genes shared by the clusters *p* and *q*, and *n*_*1*_ and *n*_*2*_ are respectively the number of marker genes associated with clusters *p* and *q*. Second, the similarity between clusters *p* and *q* is quantified by the cosine similarity of their average expression level of the union of their marker genes. Finally, the similarity between clusters *p* and *q* (i.e., the weight of the edge connecting vertices *p* and *q* in the bipartite graph) is defined as the average of the similarity values obtained from these two strategies. A pair of shared cell clusters between two datasets is identified if their similarity is greater than a threshold value *T* (0.6 by default). Note that clustering matching is performed on the confident cells, and it is possible that cluster *p* is shared by more than one cluster.

#### Learning the confounding matrix

The confounding matrix *Y* containing the information of technical variation is constructed based on all the shared cell clusters between any pair of datasets as follows. Let $\hat{X}$ represent the *n* × *m* stacked data matrix from all datasets, where *n* is the total number of confident cells and *m* is the number of selected features. ${\hat{X}}_i^p$ and ${\hat{X}}_j^q$ respectively represent the submatrices for cluster *p* in dataset *i* and cluster *q* in dataset *j*. The number of confident cells in clusters *p* and *q* are *n*_*p*_ and *n*_*q*_, respectively. Suppose that clusters *p* and *q* are a pair of shared cell clusters between dataset *i* and dataset *j*. Any differences between them are considered as technical variation (i.e., batch effects). Thus the goal is to find a set of *m*-dimensional vectors, denoted by a matrix *V* with *m* rows, to minimize the difference between the projected data ${\hat{X}}_i^pV$ from cluster *p* and ${\hat{X}}_j^qV$ from cluster *q*. For all the identified pairs of shared cell clusters between any pair of dataset *i* and dataset *j*, we minimize the technical variation after the projection by solving the following optimization problem

1 $$ \underset{V}{\max }-{\sum}_{i,j,p,q}{V}^T{\left({\hat{X}}_i^p-{\hat{X}}_j^q\ \right)}^T\left({\hat{X}}_i^p-{\hat{X}}_j^q\right)V $$

Note that we randomly subset one of the two matrices ${\hat{X}}_i^p$ and ${\hat{X}}_j^q$ to compromise the calculation of $\left({\hat{X}}_i^p-{\hat{X}}_j^q\right)$ if the two matrices have different number of rows. In order to carry out the calculation of $\left({\hat{X}}_i^p-{\hat{X}}_j^q\right)$ where the two submatrices may have different dimensions, we randomly select the cells in the larger submatrix to ensure the two submatrices have the same number of rows. Because these cells are confident cells from a pair of shared clusters with very *similar* genomic profiles across datasets, such random sampling has no or little influence on the integration performance. The inferred vectors *V* are not sensitive to the ordering of the cells. Since the expression profiles of the confident cells in each pair of clusters are similar, changing the cell ordering in ${\hat{X}}_i^p\mathrm{and}\ {\hat{X}}_j^q$ will only lead to a random noise perturbation of ${\hat{X}}_i^p-{\hat{X}}_j^q$. Of note, the optimization problem in Eq. (1) is the classic principal component analysis (PCA). Since we are interested in the inferred vectors with larger variance and PCA is known as an effective tool for random noise attenuation [48], such random noise perturbation likely has little influence on the inferred vectors.

To represent the above objective function as a matrix form for better connecting the input matrix and confounding matrix, we next reformulate the above objective function as

2 $$ \underset{V}{\max }-{V}^T{\hat{X}}^TY{Y}^T\hat{X}V. $$

Let *n*_*pq*_ = min (*n*_*p*_, *n*_*q*_), ${I}_i^p$ and ${I}_j^q$ respectively represent the sets of row indexes of cells from clusters *p* and *q* in $\hat{X}$. Then *Y* is defined as

$$ Y={\sum}_{p,q}{Y}^{pq}, $$

where each element of matrix *Y*^*pq*^ is defined as

$$ {Y}_{k,t}^{pq}=\left\{\begin{array}{c}1,k\in {I}_i^p,t\in \left\{1,2,\cdots, {n}_{pq}\right\}\\ {}-1,k\in {I}_j^q,t\in \left\{1,2,\cdots, {n}_{pq}\right\}\\ {}0, others\end{array}\right. $$

*Y*^*pq*^ is an index matrix with its elements being 1, −1, or 0 and each row of *Y*^*pq*^ contains at most one non-zero element. *Y* has *n* rows and *l* columns, where *l* is the maximum value of *n*_*pq*_ across all pairs of shared cell clusters between any pair of datasets.

#### Learning the correction vectors

After modeling the technical variation across different datasets, we now seek to learn the correction vectors by explicitly removing the technical variation [49]. This is achieved by subtracting the technical variation from the total variation after projection, which is formulated as the following optimization problem

3 $$ \underset{V}{\max }{V}^T{\hat{X}}^T\hat{X}V-{\lambda V}^T{\hat{X}}^TY{Y}^T\hat{X}V $$

$$ \mathrm{subject}\ \mathrm{to}\ {\left\Vert {V}_{,i}\right\Vert}_2^2\le 1. $$

where *V*_,*i*_ is the *i*th column of *V* and represents a *m*-dimensional correction vector. *λ* is the parameter controlling the strength of subtracting the technical variation (By default, *λ* = 10). This optimization problem can be solved by calculating eigenvectors associated with the real positive eigenvalues of ${\hat{X}}^T\hat{X}-\lambda {\hat{X}}^TY{Y}^T\hat{X}$.

The ratio of technical variation among the total variation is calculated by

4 $$ R=\frac{V^T{\hat{X}}^TY{Y}^T\hat{X}V}{V^T{\hat{X}}^TY{Y}^T\hat{X}V+{V}^T{\hat{X}}^T\hat{X}V} $$

##### Construction of corrected data for downstream analysis

*X* denotes the *N* × *m* stacked data matrix from all datasets, where *N* is the total number of cells and *m* is the number of selected features. The corrected data *L* is obtained by projecting the stacked data matrix with all cells onto the learned correction vectors *V*, i.e., *L = XV*, where each column of *V* represents a correction vector. Obviously, *L* represents a shared embedding of cells across all datasets after removing technical variation. Therefore, *L* can be used for various downstream analysis of the combined data, such as low-dimensional visualization, cell clustering, and pseudotemporal trajectory inference.

Note that the correction vectors are not cluster specific, and they are learned by subtracting the total technical variation from the total variance. If a cluster in one dataset does not match any clusters in any other datasets, we assume that this cluster is dataset-specific one. The variation of the cells in this cluster is driven by both batch effects and biological difference. Using the learned correction vectors from other shared clusters among different datasets, scMC then removes batch effects of the cells in this dataset-specific cluster by projecting the single-cell data onto these correction vectors.

##### scMC parameters

In scMC algorithm, there are two tuning parameters: *λ* and *T*. The default values for those parameters are as follows: *λ* = 10 and *T* = 0.6. scMC was found to be relatively robust to *λ* and *T* values within certain ranges (Additional file 1 and Additional file 2: Figure S16-S19).

### Method comparisons

We compared the performance of integration with three other methods, including LIGER [8], Seurat V3 [9], and Harmony [10]. LIGER (version 0.5.0.9) was run with the default parameters (*k* = 20 and lambda = 5) according to its tutorial (https://github.com/MacoskoLab/liger/blob/master/vignettes/walkthrough_pbmc.html), and it returns a shared factor matrix as a joint embedding for cells across batches. The HVGs are determined using selectGenes function. Seurat V3 (version 3.0.1) is run according to its tutorial (https://satijalab.org/seurat/v3.1/immune_alignment.html), and it returns a corrected gene expression matrix. Harmony (version 1.0) was run according to its tutorial (http://htmlpreview.github.io/?https://github.com/immunogenomics/harmony/blob/master/docs/SeuratV3.html), and it returns a shared embedding of cells. For both Seurat V3 and Harmony, HVGs are determined using the “FindVariableFeatures” function from Seurat package. We visualized cells in a two-dimensional space by applying the UMAP algorithm [22] to the corrected data outputted from each integration method. Methods were run on a macOS machine equipped with 3.2 GHz Intel Xeon W processors and 64 GB of memory.

### Evaluation metrics

To evaluate the performance of different integration methods, we use 9 metrics defined in a recent benchmarking study [19]. These 9 metrics are grouped into two categories on batch effect removal and biological variation conservation. Batch effect removal is assessed by 5 metrics, including PCR batch, batch ASW, graph iLISI, kBET, and graph connectivity [19]. Biological variation conservation, which can be captured based on the cell identity labels, is measured by 4 metrics, including label ASW, isolated label F1, isolated label silhouette, and graph cLISI. Detailed descriptions of these metrics are available in the previous study [19]. These metrics are computed using the scIB.metrics.metrics function in the excellent tool scIB (https://github.com/theislab/scib). kNN graphs are computed using scanpy.pp.neighbors function (n_neighbors = 20), where Euclidean distances were computed on the top 40 PCs of the corrected feature matrix.

In addition, we compute another 7 biological conservation metrics, including NMI label, ARI label, Jaccard label, Purity label, silhouette score, specificity score, and LISI-derived F1 score. The first four metrics are used to quantitatively assess the accuracy of inferred cell subpopulations from the corrected data, and the last three metrics are used to assess biological conservation by quantifying the separation of cell types, how less overlap between the condition-specific cell types with other cells, and both batch mixing and cell type separation in the shared UMAP space. Moreover, we define overall accuracy scores by averaging the rank of each integration method based on three categories of metrics, including batch effect removal metrics, biological conservation metrics, and both batch effect removal and biological conservation metrics, respectively. Assume that *m*_1_ batch effect removal metrics and *m*_2_ biological conservation metrics are computed for each method. For each metric in the batch effect removal category, the four methods are arranged in a decrease order based on the computed matric scores and assigned with values from 4 to 1. For all the *m*_1_ metric computed for each method, we compute the average assigned values as the overall score for batch effect removal. Similarly, we compute an overall score for each method using the *m*_2_ biological conservation metrics. Finally, we compute an overall score for each method by using both batch effect removal and biological conservation metrics (i.e., *m*_1_ + *m*_2_ metrics). Thus, a larger overall accuracy score indicates better performance.

#### NMI label, ARI label, Jaccard label, and Purity label

We quantified the accuracy of inferred cell subpopulations from the corrected data using four metrics, including normalized mutual information (NMI) [50], Adjusted Rand Index (ARI) [51], Jaccard index, and Purity. We used these metrics to compare the true cell labels with the Leiden clusters identified from the corrected data. All these metrics range from 0 to 1, with the higher value representing the better clustering. NMI is a variation of mutual information for measuring clustering accuracy. ARI quantifies the concordance between two clustering results. A value of 0 or 1 corresponds to random clustering or a perfect match, respectively. The Jaccard index quantifies the overlap between two clustering results. A value of 0 or 1 indicates no common element or identical elements between the two clustering results, respectively. Purity quantifies the extent to which a cluster contains a single class.

#### Silhouette score

Silhouette score assesses the separation of cell types in a low-dimensional space. A high score suggests that cells of the same cell type are close together and far from other cells of a different type. The silhouette score of a cell *i* is defined as [52]

5 $$ s(i)=\frac{b(i)-a(i)}{\max \left(a(i),b(i)\right)} $$

$$ a(i)=\frac{1}{\left|{C}_i\right|}\sum \limits_{\forall j}d\left({x}_i,{x}_j\right),b(i)=\underset{\forall j,j\notin {C}_i}{\min }d\left({x}_i,{x}_j\right) $$

where *a(i)* is the average distance of cell *i* to all other cells within *i*’s cluster and *b(i)* is the average distance of *i* to all cells in the nearest cluster to which *i* does not belong. Then the overall silhouette score equals the average values of all cells.

#### LISI-derived F1 score

LISI is used to assess the performance of batch and cell type mixing [10]. LISI selects the nearest neighbors based on the local distances with a fixed perplexity and uses these selected nearest neighbors to compute inverse Simpson’s index for batch (bLISI) or cell type (cLISI) labels. For each cell in the dataset, the bLISI and cLISI scores are computed and normalized by (median (scores) − min (scores))/(max (scores) − min (scores)). To assess both batch mixing and cell type separation, we summarize these two metrics into a single F1 score using a similar formula in a previous study [13]. We term this new score as a LISI-derived F1 score, which is defined as:

6 $$ \mathrm{LISI}-\mathrm{derived}\ \mathrm{F}1=\frac{2\mathrm{bLISI}\left(1-\mathrm{cLISI}\right)}{1-\mathrm{cLISI}+\mathrm{bLISI}} $$

#### Specificity score

This metric quantifies how less overlap between the condition-specific cell types with other cells in a low-dimensional space. The higher specificity score indicates the less overlap. In a low-dimensional space shared by different conditions, we build a *k*-nearest neighbor graph (*k* = 20 by default). For each cell in the condition-specific cell types, we count how many of its nearest neighbors (denoted by *n*) are the cells that are not in the specific cell types. Then the overlap ratio of each cell in the specific cell types with other cells is computed by *n/k*. Finally, the specificity score equals one minus the median value of the computed ratios from all cells. Of note, the specificity score is different from the aforementioned cLISI metric. The specificity score evaluates the level of preserving dataset-specific cell types, whereas cLISI evaluates the preservation of all cell types. If there are dataset-specific cell types across different datasets, both bLISI and cLISI are unable to evaluate whether the integration method can preserve such specific cell types.

#### Cell pseudotime conversation score

We inferred the pseudotime from the corrected data of each method using Monocle 2 [53]. The pseudotime consistency between the inferred pseudotime and the gold standard pseudotime (i.e., ground truth) was evaluated by two metrics: pseudotime ordering score (*POS*) and Kendall rank correlation coefficient. The *POS* is defined as [54]

7 $$ \mathrm{POS}=\frac{\mathrm{C}}{{\mathrm{N}}_{\mathrm{C}}+\mathrm{C}} $$

where *C* represents the number of similar orders between the pseudotime and gold standard orders, and *N*_*c*_ denotes the number of dissimilar orders.

For the mouse skin embryonic development scRNA-seq dataset, the gold standard trajectories and pseudotime are lacking. To compare the recovered trajectories in a quantitative fashion, we use a similar method from Luecken et al. [19], which assumes that trajectories inferred using the unintegrated data for each batch are the more accurate biological signal. For the unintegrated data, we compute the pseudotime values of cells by analyzing each replicate sample separately and then calculate Pearson correlation coefficients between the pseudotime values of cells in each replicate sample before and after integration. To infer the pseudotime values of cells, we adopt a principal curve-based approach used in the previous pseudotime-inference tools such as scEpath [31] and Slingshot [55]. Specifically, we fit a principal curve in the PHATE space and obtain the pseudotime values by orthogonal projections of cells onto the curves.

## Supplementary Information

**Additional file 1.** Supplementary Methods.

**Additional file 2.** Supplementary Figures; Figure S1-S19.

**Additional file 3.** Review history.

## Data Availability

*Codes*: scMC is publicly available as an R package under the GPL-3 license. Source codes, tutorials, and reproducible benchmarking codes have been deposited at the GitHub repository (https://github.com/amsszlh/scMC) [56] and Zenodo repository (DOI: 10.5281/zenodo.4138819) [57]. *Datasets*: The control and interferon *β*-stimulated human PBMC scRNA-seq datasets [23], including 13,999 cells across two batches, were accessed through the SeuratData package (https://github.com/satijalab/seurat-data). The control and Hedgehog activation mouse skin scRNA-seq datasets, including 4680 cells across two batches, were accessed from the Gene Expression Omnibus (GEO) database under accession code GSE112671 [25]. The skin embryonic development scRNA-seq dataset, including 25,719 cells across four batches collected from two time points, was accessed from GEO database under accession code GSE122043 [29]. The scATAC-seq dataset, including 4451 cells, was accessed from GEO database under accession code GSE111586 [34]. The mouse lung scRNA-seq dataset consisting of 32,472 cells across 16 batches was downloaded from https://figshare.com/authors/Malte_Luecken/8928848.
