# Supplementary material for: scMC learns biological variation through the alignment of multiple single-cell genomics datasets
Source: Genome Biol. 2021 Jan 4;22:10. doi: 10.1186/s13059-020-02238-2 (PMC7784288; doi:10.1186/s13059-020-02238-2)
Supplement: Supplementary file 1 — Additional file 1. Supplementary Methods. [file 13059_2020_2238_MOESM1_ESM.pdf]

Additional file 1: Supplementary Methods for

scMC learns biological variation through the alignment of multiple single-cell genomics datasets

Lihua Zhang[1,2] and Qing Nie[1,2,3,#]

[1] Department of Mathematics, University of California, Irvine, CA 92697, USA

[2] NSF-Simons Center for Multiscale Cell Fate Research, University of California, Irvine, CA 92697, USA

[3] Department of Developmental and Cell Biology, University of California, Irvine, CA 92697, USA

Supplementary Methods

Simulation datasets

Simulation dataset 1. Dataset 1 contains two batches with 2000 cells in each batch. When generating this dataset using Splatter package, the factor Scale of each batch equals 0.1 and 0.2. Each batch has four cell groups with parameter *group.prob* equaling (0.1, 0.2, 0.3, 0.4). The detailed parameters were as follows:

Sim1 <- splatSimulate(batchCells = c(2000, 2000), batch.facScale = c(0.1, 0.2), group.prob = c(0.1, 0.2, 0.3, 0.4), method = “groups”, verbose = FALSE)

Simulation dataset 2. Compared to dataset 1, there are imbalanced cell subpopulation compositions between the two batches in dataset 2. We added two more cell clusters to the first batch of dataset 1 as follows:

Sim2 <- splatSimulate(batchCells = 1000, group.prob = c(0.5, 0.5), method = “groups”, verbose = FALSE)

In sum, dataset 2 has two batches with six cell clusters in one batch and four cell clusters in another batch.

Simulation dataset 3. Dataset 3 contains three batches with each batch has 1000, 1000, 2000 cells. We first generated balanced cell clusters for all batches, and then removed one cluster from the first batch and two clusters from the third batch. The dataset with balanced cell clusters was generated by:

Sim4 <- splatSimulate(batchCells = c(1000, 1000, 2000), batch.facScale = c(0.1, 0.2, 0.3), group.prob = c(0.1, 0.2, 0.2, 0.2, 0.3), method = “groups”, verbose = FALSE)

Dataset 3 was obtained after removing group 2 from batch 1 and group 3 and group 5 from batch 3. In sum, dataset 3 has four clusters, five clusters and three clusters in batch 1, 2, 3.

Simulation dataset 4. Dataset 4 describes a continuous cell trajectory across two batches with 1000 cells per batch. The detailed parameters were shown as follows:

Sim3 <- splatSimulate(batchCells = c(1000, 1000), batch.facScale = c(0.1, 0.2), method = “paths”, verbose = FALSE)

Simulation dataset 5. Dataset 5 consists of 12,097 cells with six batches and seven cell groups, which was simulated by Splatter package using the codes provided in https://github.com/theislab/scib/blob/master/notebooks/data_preprocessing/simulations/sim1.R. Detailed information can be found in Luecken et al[1].

Simulation dataset 6. Dataset 6 consists of 19,318 cells with 16 nested sub-batches (four sets of four sub-batches) and 4 cell groups, which was simulated by Splatter package using the codes provided in https://github.com/theislab/scib/blob/master/notebooks/data_preprocessing/simulations/sim2.R. Detailed information can be found in Luecken et al[1].

Simulation datasets for evaluating running time. We created five simulated datasets with 1,000, 5,000, 10,000, 20,000 and 30,000 cells, to evaluate the running time. Each dataset contains three batches and five clusters. For example, for the dataset with 20,000 cells, the detailed parameters were as follows:

sim <- splatSimulate(batchCells = c(6000, 4000, 10000), batch.facScale = c(0.1,0.2,0.3),group.prob = c(0.1,0.2,0.2,0.2,0.3), method = "groups", verbose = FALSE)

Robustness analysis of tuning parameters

In scMC, there are two tuning parameters: λ and T. λ controls the relative contribution of the technical variation when learning correction vectors. When λ increases, the ratio between the technical variation and the total amount of variation becomes stabilized (see Methods in main text, Additional file 2 Figure S16B, S17B). $\lambda = 10$, which is used as a default value, usually provides a stable result. scMC was found to be relatively robust when λ was greater than a certain value (Additional file 2 Figure S16A, S17A). T is a thresholding parameter determining whether cell clusters are shared across different datasets based on their similarity. If T is too small, the biological variation may be removed. If T is too large, the technical variation might not be completely removed. It was found that T larger than 0.5 provides better results, with T=0.6 (as default value) used for all the datasets. By visualizing the corrected data in UMAP using both simulated and real datasets, scMC was found to be relatively robust to T values within certain ranges (Additional file 2 Figure S18-S19).

Biological function analysis of the identified cell subpopulations in the brain tissue from adult mouse scATAC-seq dataset

Four cell subpopulations in the brain tissue were identified from scMC-integrated data on the ChromVAR kmer transformed scATAC-seq data (Additional file 2 Figure S13A). To gain insights into the biological functions of these identified subpopulations, we first identified differential loci of these four cell subpopulations by aggregating scATAC-seq data of each cell subpopulation and performing Wilcox rank test on the aggregated scATAC-seq data. We aggregated scATAC-seq data of each cell subpopulation by summing the single cell chromatin profiles of randomly selected 10 cells in each cell subpopulation. Second, we identified enriched transcriptional factors (TFs) in these differential loci using chromVAR [2]. chromVAR calculates the bias corrected deviations in accessibility. For each motif, there is a value for each cell, which measures how different the accessibility for loci with that motif is from the expected accessibility based on the average of all the cells. By performing hierarchical clustering of the calculated deviations of the identified128TFs, we found that the patterns of these TFs were almost specific to each particular cell subpopulation (Additional file 2 Figure S13B). Third, we used GREAT[3] to detect enriched biological processes of the differential loci (Additional file 2 Figure S13C).

References

1. Luecken M, Büttner M, Chaichoompu K, Danese A, Interlandi M, Mueller M, Strobl D, Zappia L, Dugas M, Colomé-Tatché M, Theis F: **Benchmarking atlas-level data integration in single-cell genomics.** *bioRxiv* 2020.
2. Schep AN, Wu B, Buenrostro JD, Greenleaf WJ: **chromVAR: inferring transcription-factor-associated accessibility from single-cell epigenomic data.** *Nat Methods* 2017, **14:**975-978.
3. McLean CY, Bristor D, Hiller M, Clarke SL, Schaar BT, Lowe CB, Wenger AM, Bejerano G: **GREAT improves functional interpretation of cis-regulatory regions.** *Nat Biotechnol* 2010, **28:**495-501.
