# Supplementary material for: scMC learns biological variation through the alignment of multiple single-cell genomics datasets
Source: Genome Biol. 2021 Jan 4;22:10. doi: 10.1186/s13059-020-02238-2 (PMC7784288; doi:10.1186/s13059-020-02238-2)
Supplement: Supplementary file 2 — Additional file 2. Supplementary Figures; Figure S1-S19. [file 13059_2020_2238_MOESM2_ESM.pdf]

Additional file 2: Supplementary Figures for

scMC learns biological variation through the alignment of multiple single-cell genomics datasets

Lihua Zhang[1,2] and Qing Nie[1,2,3,#]

[1] Department of Mathematics, University of California, Irvine, CA 92697, USA

[2] NSF-Simons Center for Multiscale Cell Fate Research, University of California, Irvine, CA 92697, USA

[3] Department of Developmental and Cell Biology, University of California, Irvine, CA 92697, USA

Supplementary Figures

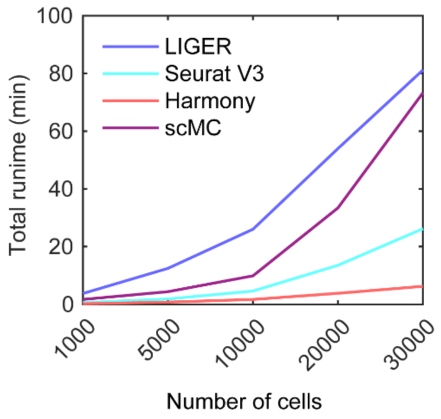

Figure S1. Comparison of total runtime required to analyze each simulated dataset. The number of cells of these simulated datasets are 1,000, 5,000, 10,000, 20,000 and 30,000.

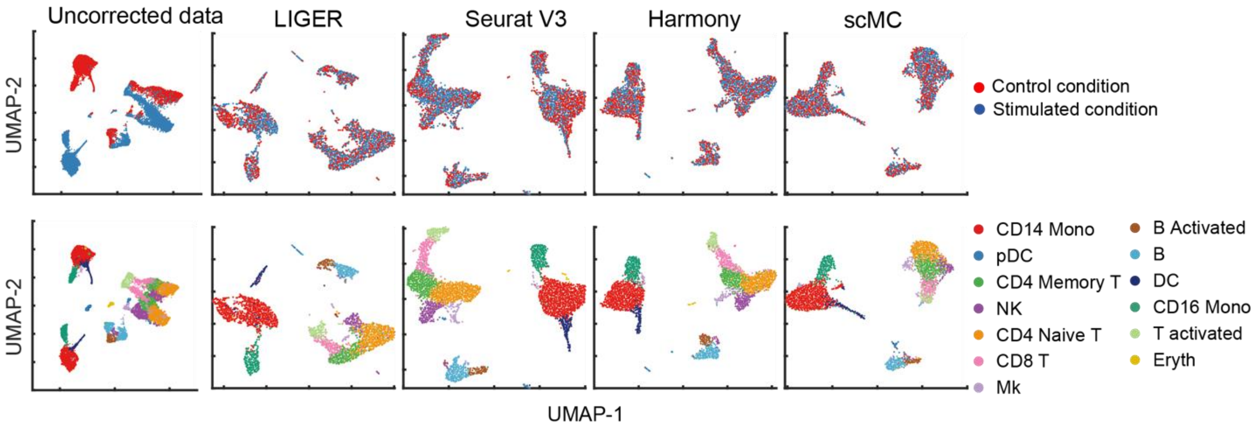

Figure S2. Comparison of integration performance of scMC with LIGER, Seurat V3 and Harmony on PBMC dataset with all cells. UMAP visualization of the uncorrected data, and the corrected data by LIGER, Seurat V3, Harmony and scMC. Cells are colored by experimental conditions (top panels). Red and blue represent control and stimulated conditions. In the bottom panels, cells are colored based on the published cell labels.

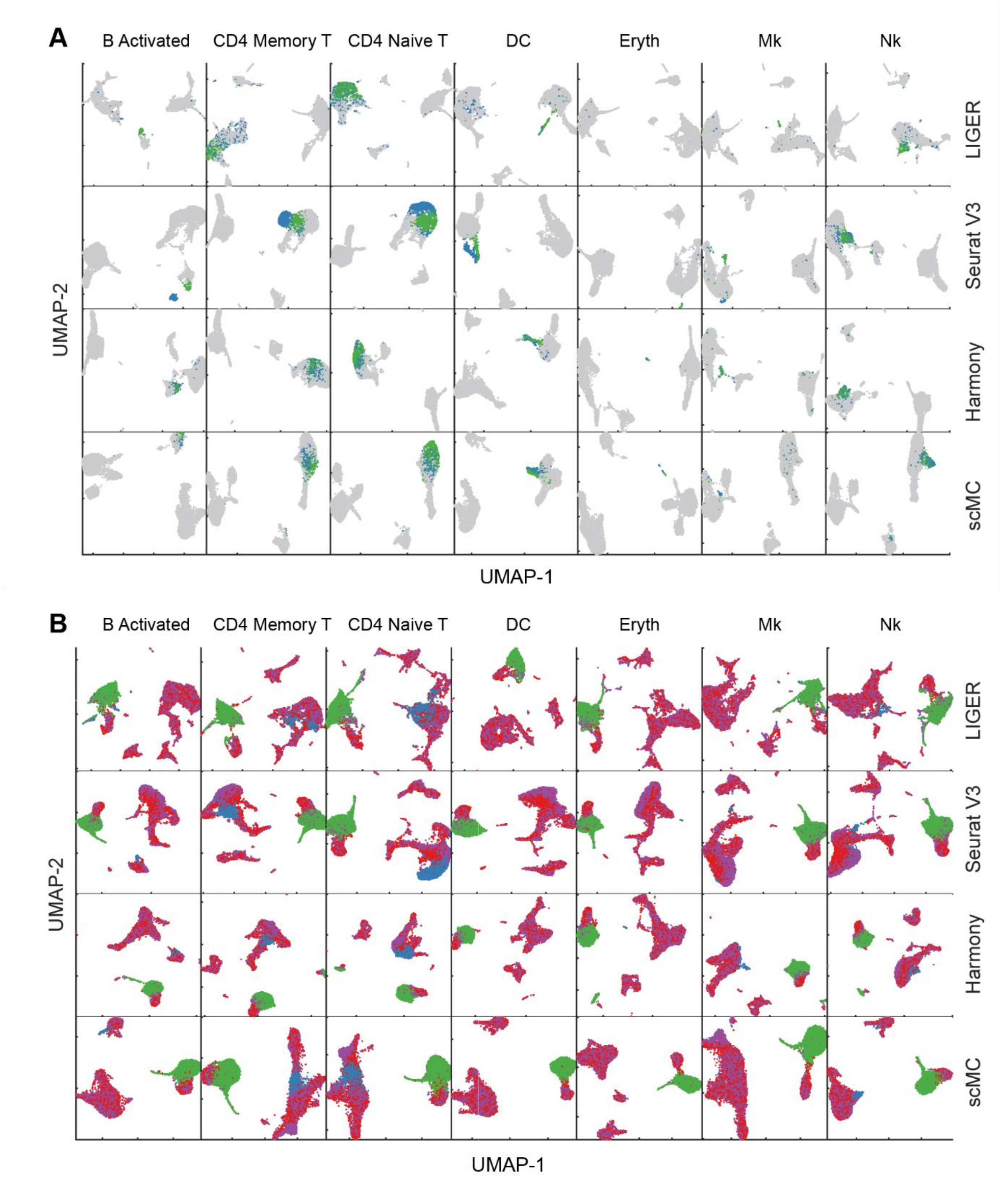

Figure S3. scMC aligns and preserves condition-specific cell subpopulations on perturbed PBMC datasets. (A) UMAP visualization of the corrected data from LIGER, Seurat V3, Harmony and scMC across the control and stimulated conditions in the perturbed PBMC datasets. Each row represents the results from one method, and each column represents one perturbed dataset in which only one cell subpopulation was retained in the control condition (indicated on the top).

Cells that are retained in the control condition were colored by green, cells from the corresponding same cell subpopulation in the stimulated condition are colored by blue, and other cells in the stimulated condition are colored by grey. **(B)** UMAP visualization of the corrected data from LIGER, Seurat V3, Harmony and scMC across the control and stimulated conditions. Each column represents one perturbed dataset, where the cell subpopulation removed in the control condition is labeled on the top, and CD14 Mono and DC cell subpopulations are also removed in the stimulated condition for all cases. CD14 Mono and DC cells from the control condition are colored by green, and other cells from the control condition are colored by red. The cell subpopulation removed from the control condition is specific in the stimulated condition, which are colored by blue. Other cells in the stimulated condition are colored by purple.

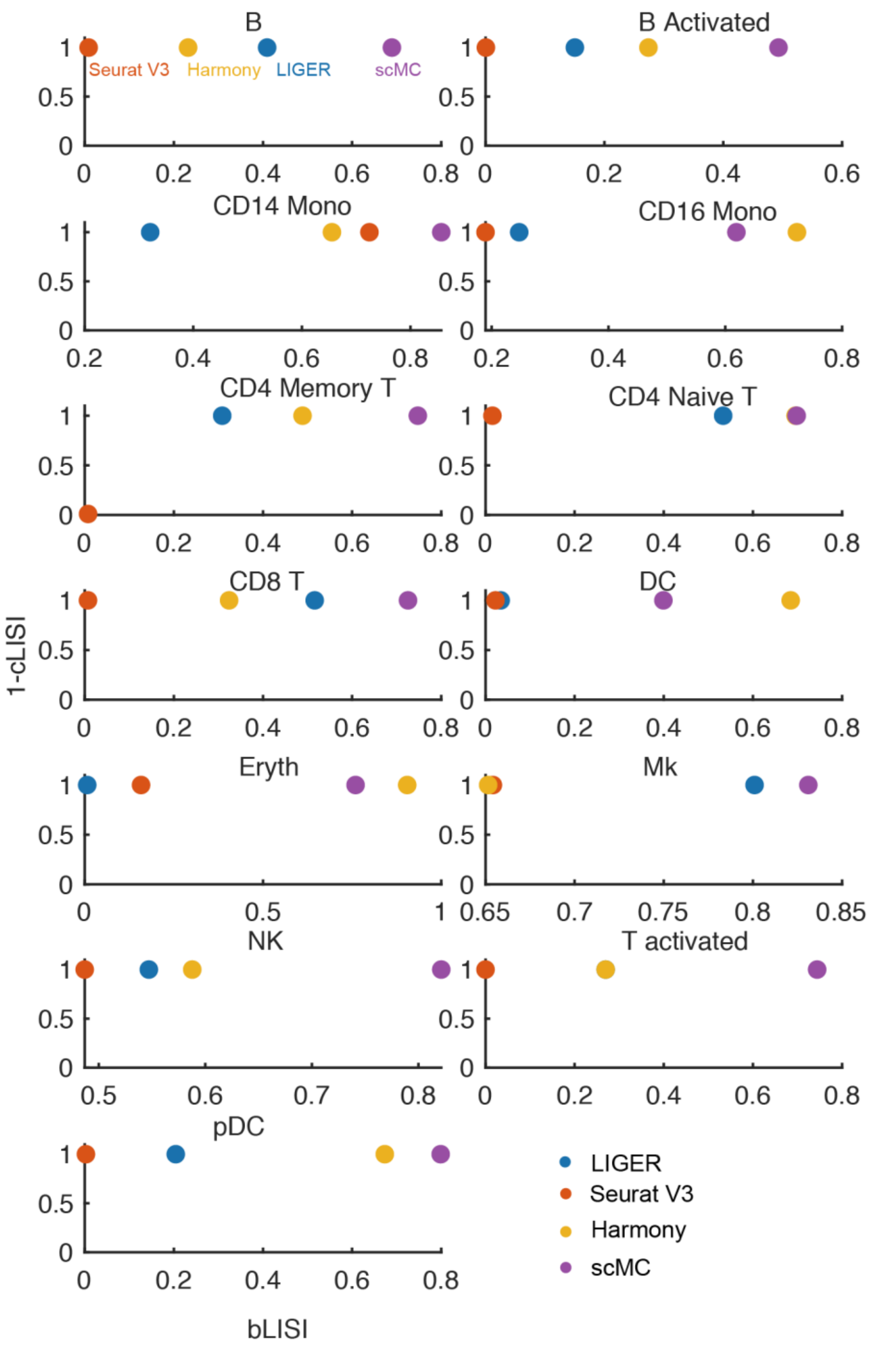

Figure S4. Comparison of integration performance by evaluating the trade-off between bLISI and cLISI on the perturbed PBMC dataset, related to Figure 3a. Each panel shows the results of one perturbed dataset in which only one cell subpopulation was retained in the control condition (indicated on the top). Each dot plot shows the computed bLISI (x-axis) and 1-cLISI (y-axis) of each method. One dot represents one method. scMC consistently exhibits better performance on both batch effect removal and cell type separation, which are assessed by bLISI and 1-cLISI.

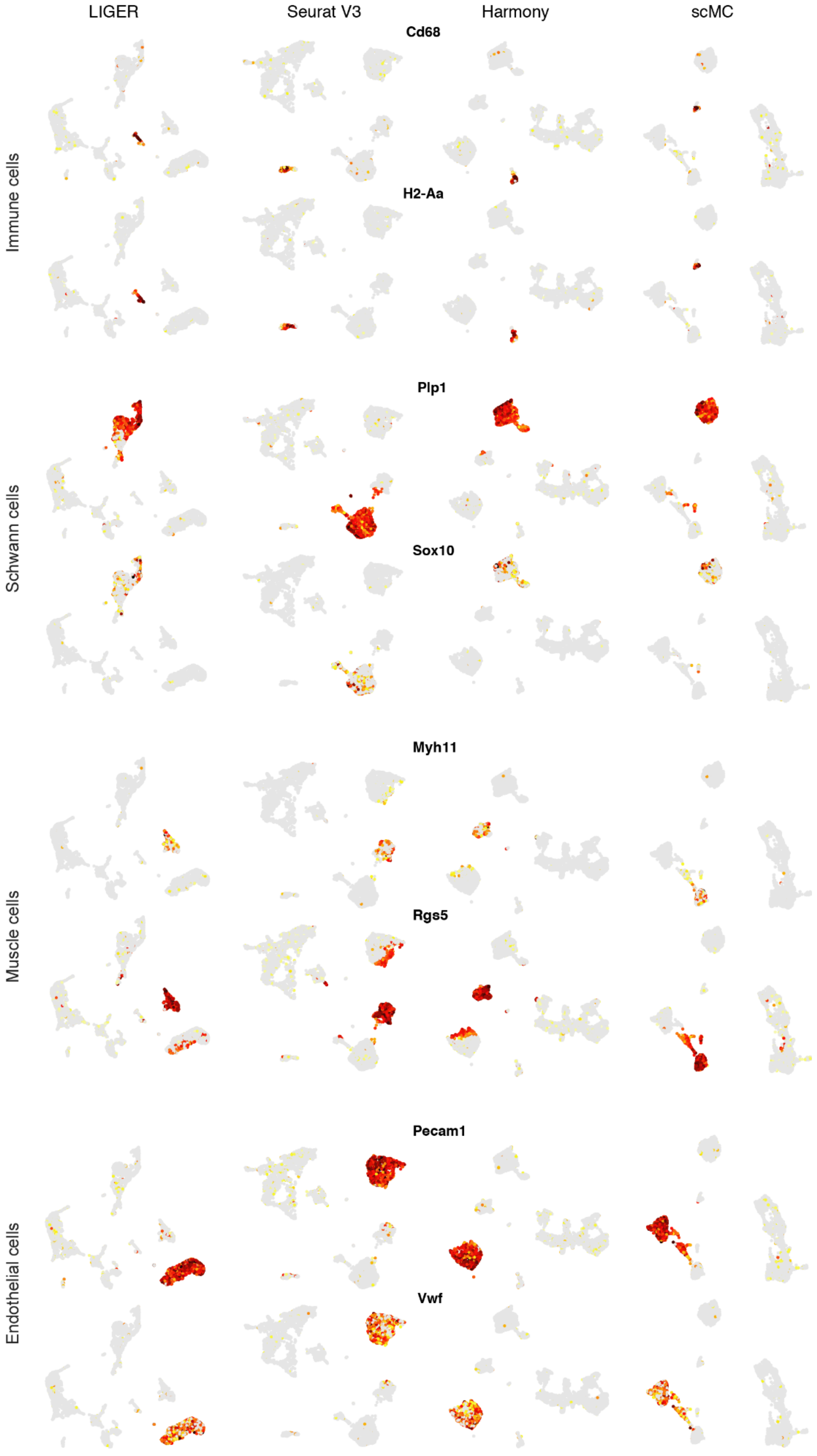
LIGER
Seurat V3
Harmony
scMC
Immune cells
Cd68
H2-Aa
Schwann cells
Plp1
Sox10
Muscle cells
Myh11
Rgs5
Endothelial cells
Pecam1
Vwf

Figure S5. Overlay the expression levels of known marker genes associated with each population in control and Hedgehog activation during mouse skin wound healing. Each column represents the UMAP visualization of the corrected data from one of the four methods: LIGER, Seurat V3, Harmony and scMC. Cells are colored based on the expression levels of each marker gene. Dark red and grey colors represent the high and zero expression.

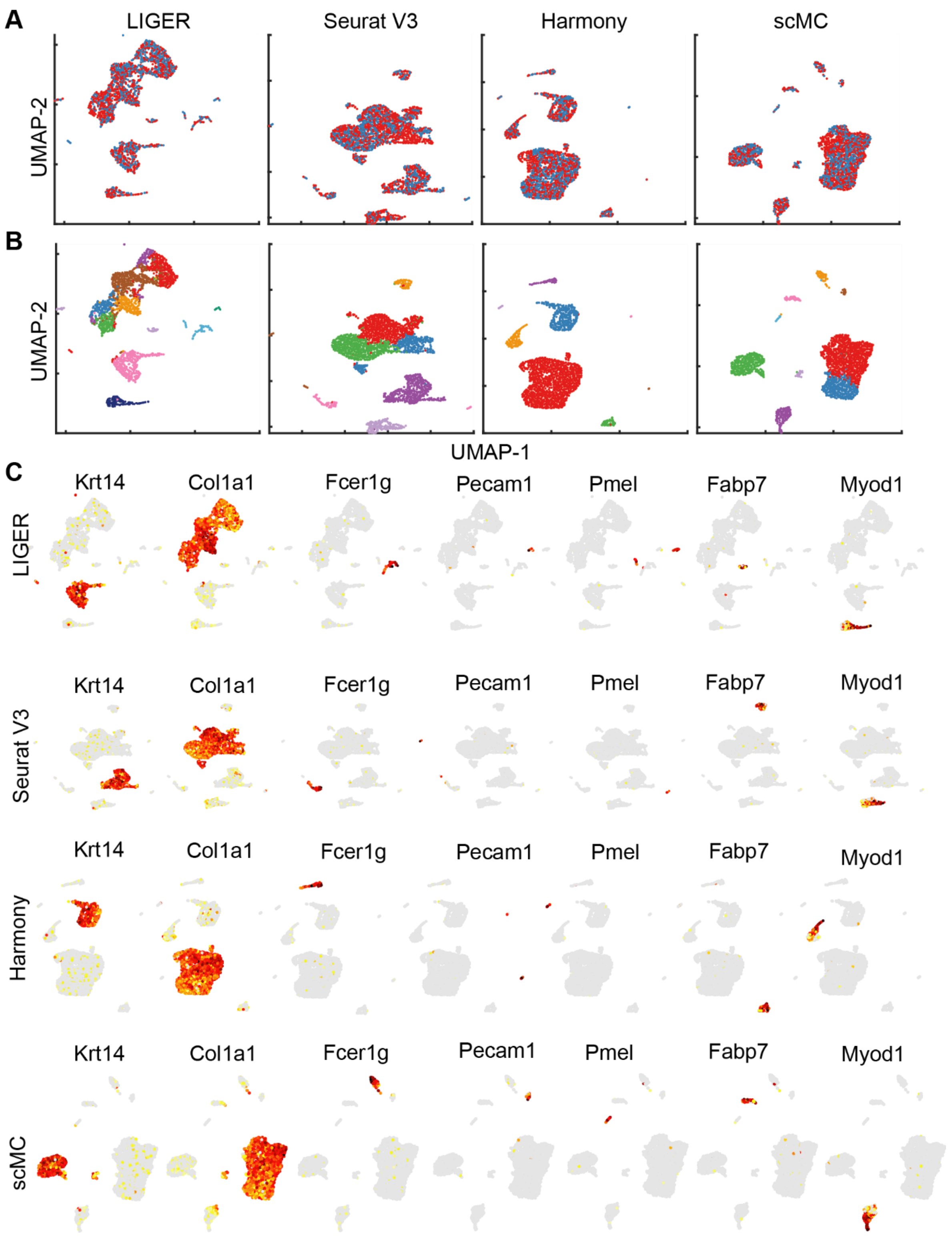

Figure S6. The performance of LIGER, Seurat V3, Harmony and scMC on the integration of two replicates from skin E13.5 embryonic development datasets. (A) UMAP visualization of

the corrected data from LIGER, Seurat V3, Harmony and scMC across the two E13.5 biological replicates. Cells are colored by replicate labels. Red and blue colors represent replicates 1 and 2. **(B)** UMAP visualization of the corrected data from LIGER, Seurat V3, Harmony and scMC across the two E13.5 biological replicates. Cells are colored based on the identified cell subpopulations by applying Leiden algorithm to the corrected data of each method. **(C)** Overlay the expression levels of marker genes onto the UMAP spaces given by LIGER, Seurat V3, Harmony and scMC. Each row represents the UMAP space of one method. Dark red and grey colors represent the high and zero expression.

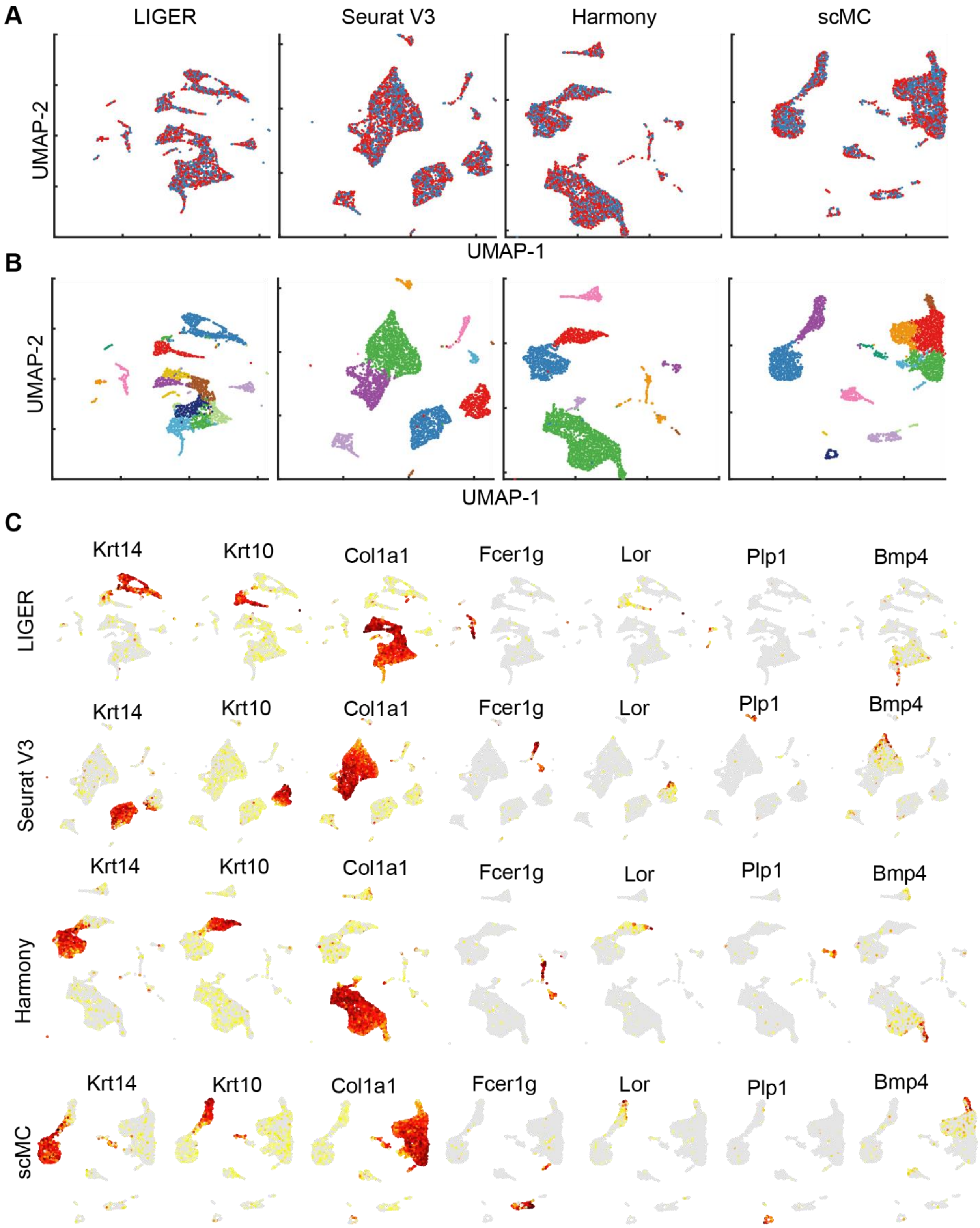

Figure S7. The performance of LIGER, Seurat V3, Harmony and scMC on the integration of two replicates from skin E14.5 embryonic development datasets. (A) UMAP visualization of

the corrected data from LIGER, Seurat V3, Harmony and scMC across the two E14.5 biological replicates. Cells are colored by replicate labels. Red and blue colors represent replicates 1 and 2. **(B)** UMAP visualization of the corrected data from LIGER, Seurat V3, Harmony and scMC across the two E14.5 biological replicates. Cells are colored based on the identified cell subpopulations by applying Leiden algorithm to the corrected data of each method. **(C)** Overlay the expression levels of marker genes onto the UMAP spaces given by LIGER, Seurat V3, Harmony and scMC. Each row represents the UMAP space of one method.

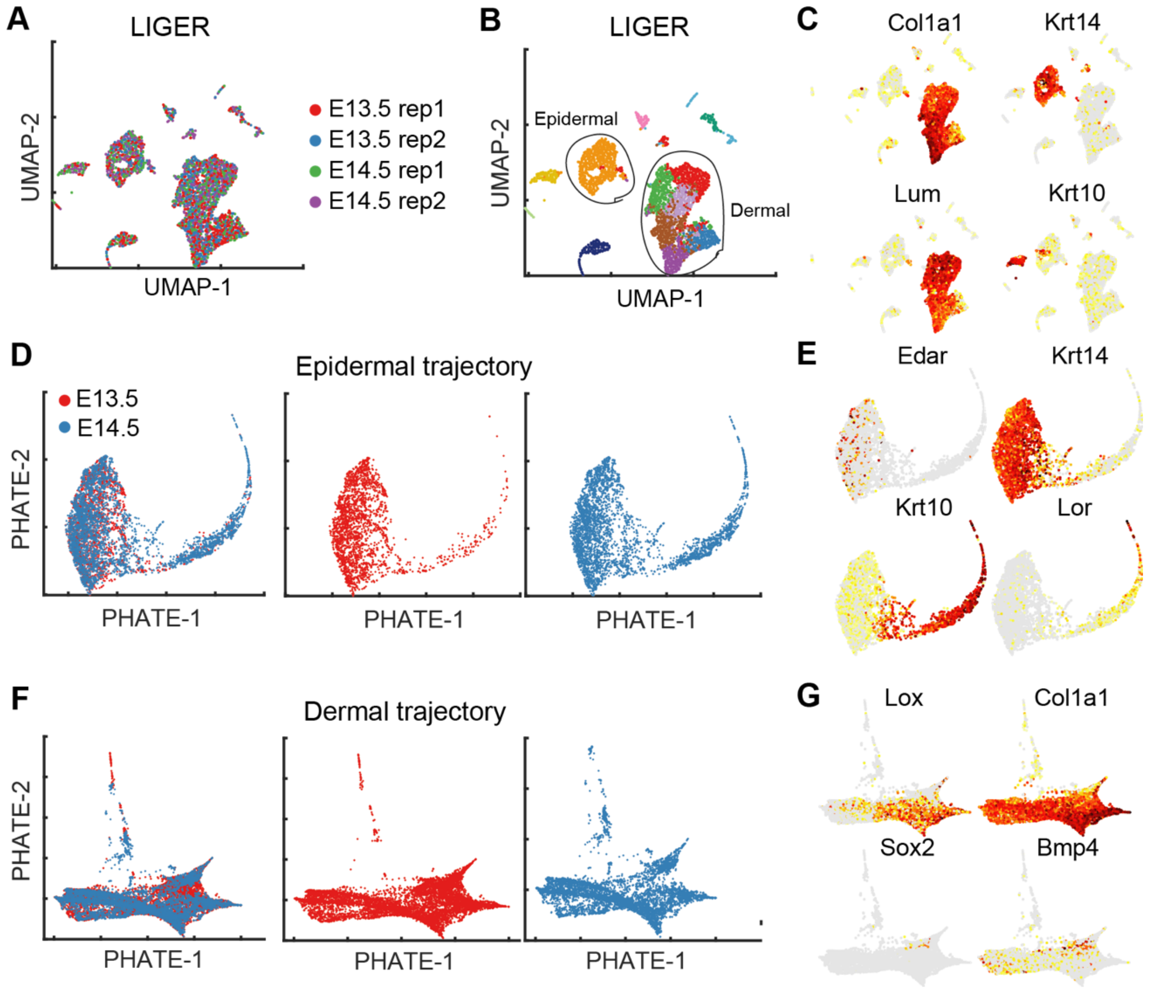

Figure S8. The performance of LIGER on the integration of single cell time course data during skin embryonic development. **(A)** UMAP visualization of the corrected data from LIGER on the time course scRNA-seq datasets from E13.5 to E14.5. Cells are colored by the replicates and time points. **(B)** UMAP visualization of the corrected data from LIGER. Cells are colored by the identified cell subpopulations from the corrected data. **(C)** Overlay the expression levels of

markers of dermal (Col1a1 and Lum) and epidermal cells (Krt14 and Krt10) onto the UMAP space. **(D)** PHATE visualizations for the epidermal cells from both E13.5 and E14.5, only E13.5 and only E14.5. **(E)** Overlay the expression levels of markers of epidermal cells (Krt5, Krt14, Krt10 and Lor) onto the PHATE space. **(F)** PHATE visualizations for the dermal cells from both E13.5 and E14.5, only E13.5 and only E14.5. **(G)** Overlay the expression levels of markers of dermal cells (Lox and Col1a1) and DC cells (Sox2 and Bmp4) onto the PHATE space.

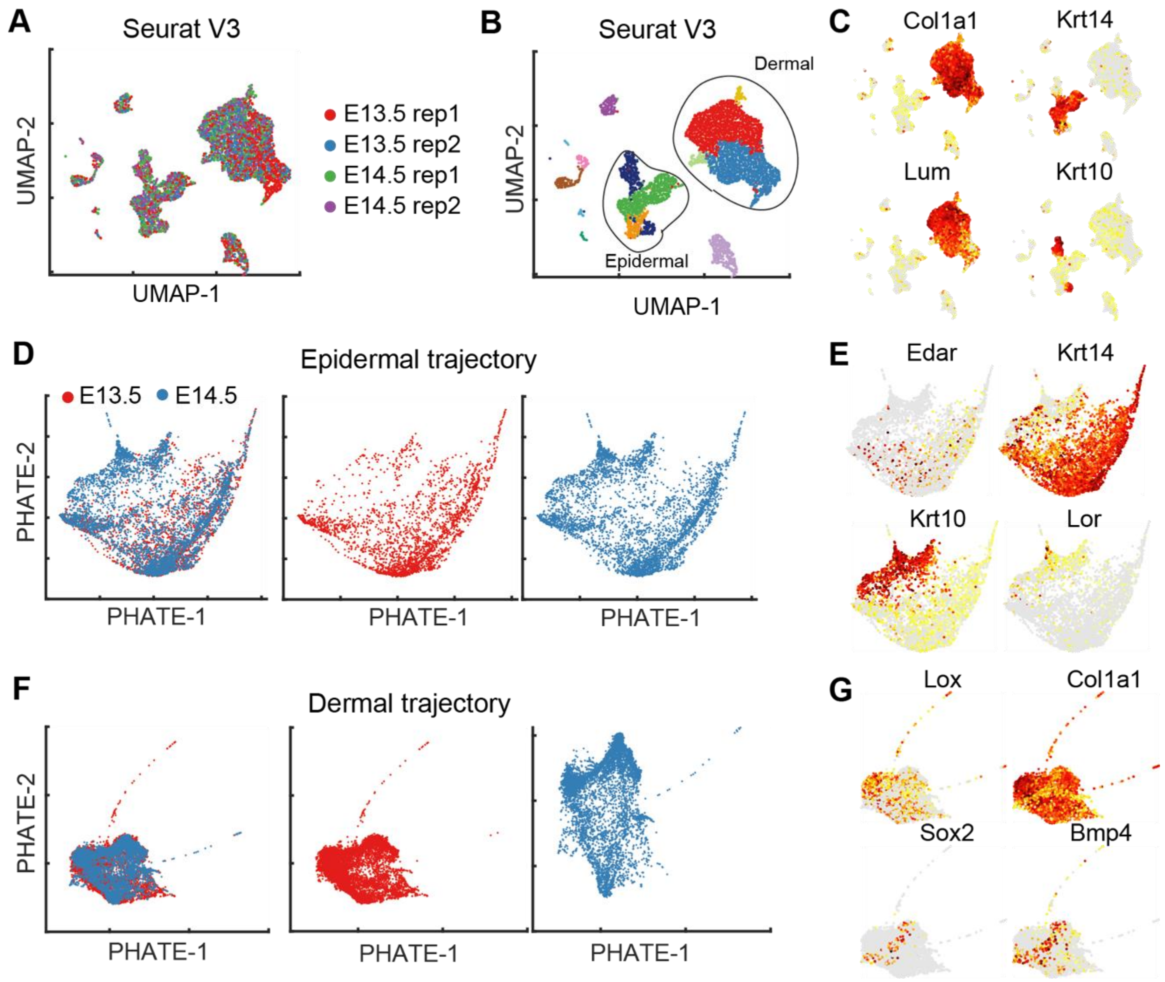

Figure S9. The performance of Seurat V3 on the integration of single cell time course data during skin embryonic development. (A) UMAP visualization of the corrected data from Seurat V3 on the time course scRNA-seq datasets from E13.5 to E14.5. Cells are colored by the replicates and time points. **(B)** UMAP visualization of the corrected data from Seurat V3. Cells are colored by the identified cell subpopulations from the corrected data. **(C)** Overlay the expression levels of markers of dermal (Col1a1 and Lum) and epidermal cells (Krt14 and Krt10) onto the

UMAP space. **(D)** PHATE visualizations for the epidermal cells from both E13.5 and E14.5, only E13.5 and only E14.5. **(E)** Overlay the expression levels of markers of epidermal cells (Krt5, Krt14, Krt10 and Lor) onto the PHATE space. **(F)** PHATE visualizations for the dermal cells from both E13.5 and E14.5, only E13.5 and only E14.5. **(G)** Overlay the expression levels of markers of dermal cells (Lox and Col1a1) and DC cells (Sox2 and Bmp4) onto the PHATE space.

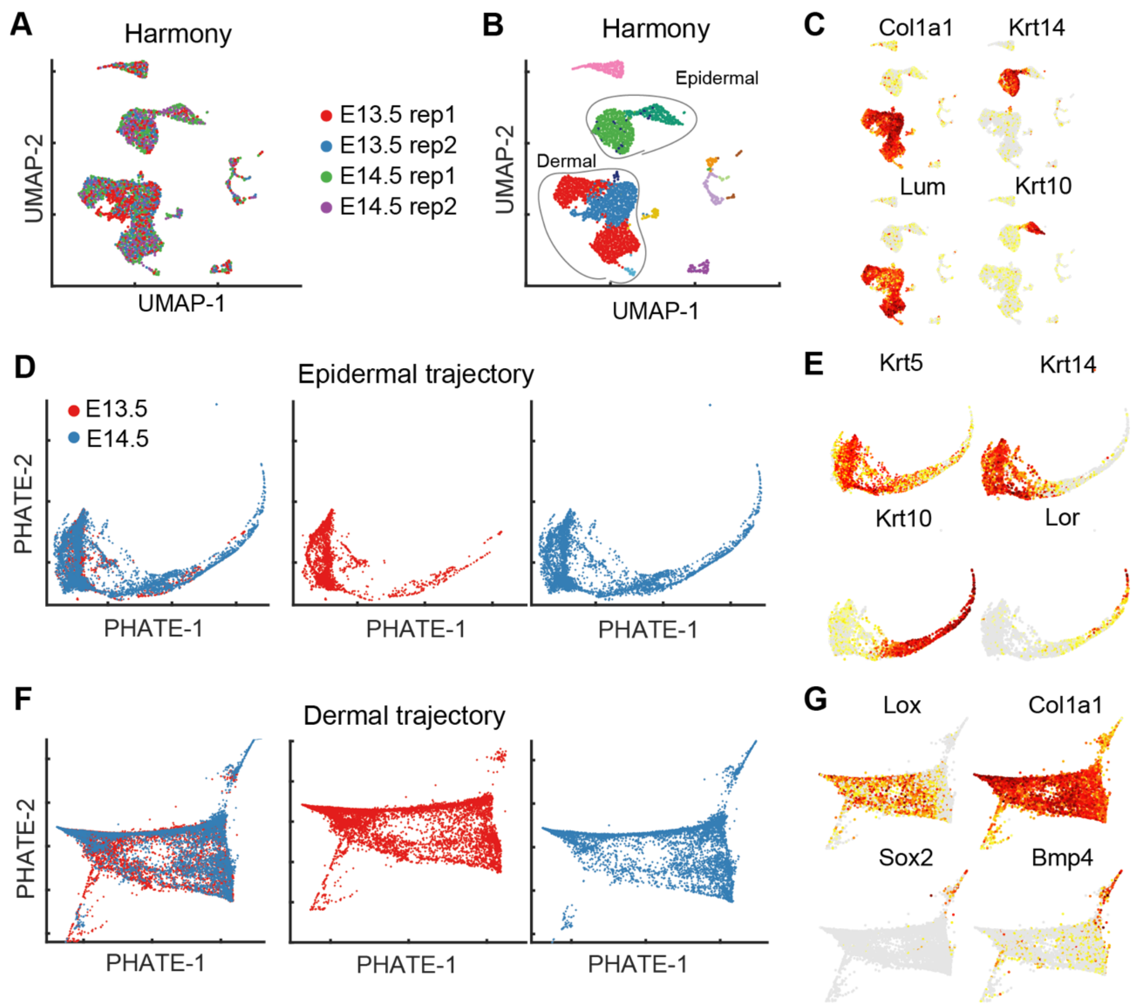

Figure S10. The performance of Harmony on the integration of single cell time course data during skin embryonic development. **(A)** UMAP visualization of the corrected data from Harmony on the time course scRNA-seq datasets from E13.5 to E14.5. Cells are colored by the replicates and time points. **(B)** UMAP visualization of the corrected data from Harmony. Cells are colored by the identified cell subpopulations from the corrected data. **(C)** Overlay the expression levels of markers of dermal (Col1a1 and Lum) and epidermal cells (Krt14 and Krt10) onto the UMAP space. **(D)** PHATE visualizations for the epidermal cells from both E13.5 and E14.5, only

E13.5 and only E14.5. **(E)** Overlay the expression levels of markers of epidermal cells (Krt5, Krt14, Krt10 and Lor) onto the PHATE space. **(F)** PHATE visualizations for the dermal cells from both E13.5 and E14.5, only E13.5 and only E14.5. **(G)** Overlay the expression levels of markers of dermal cells (Lox and Col1a1) and DC cells (Sox2 and Bmp4) onto the PHATE space.

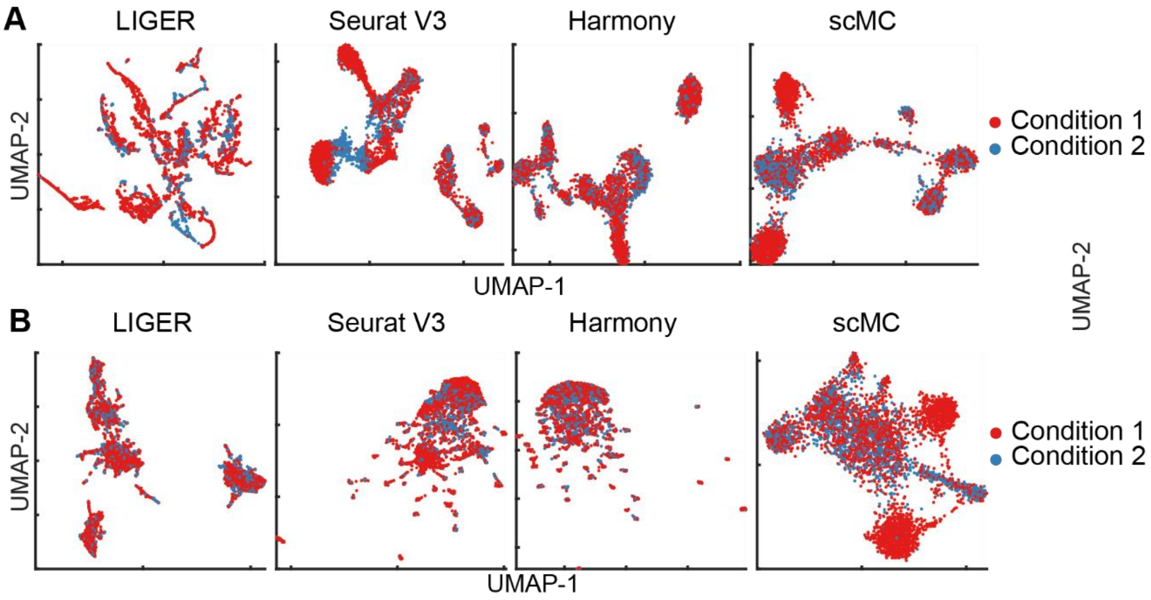

Figure S11. The integration performance of LIGER, Seurat V3, Harmony and scMC on the scATAC-seq data. (A) UMAP visualization of the corrected data from LIGER, Seurat V3, Harmony and scMC on scATAC-seq data with the feature matrix transformed by ChromVAR. Cells are colored by the biological conditions. Red color represents condition 1 with cells from Whole Brain replicate 1, Large Intestine replicate 1, Liver and Heart. Blue color represents condition 2 with cells from Whole Brain replicate 2 and Large Intestine replicate 2. **(B)** UMAP visualization of the corrected data from LIGER, Seurat V3, Harmony and scMC on scATAC-seq data with the feature matrix transformed by Gene Scoring. scMC outperformes other methods in preserving condition-specific tissues.

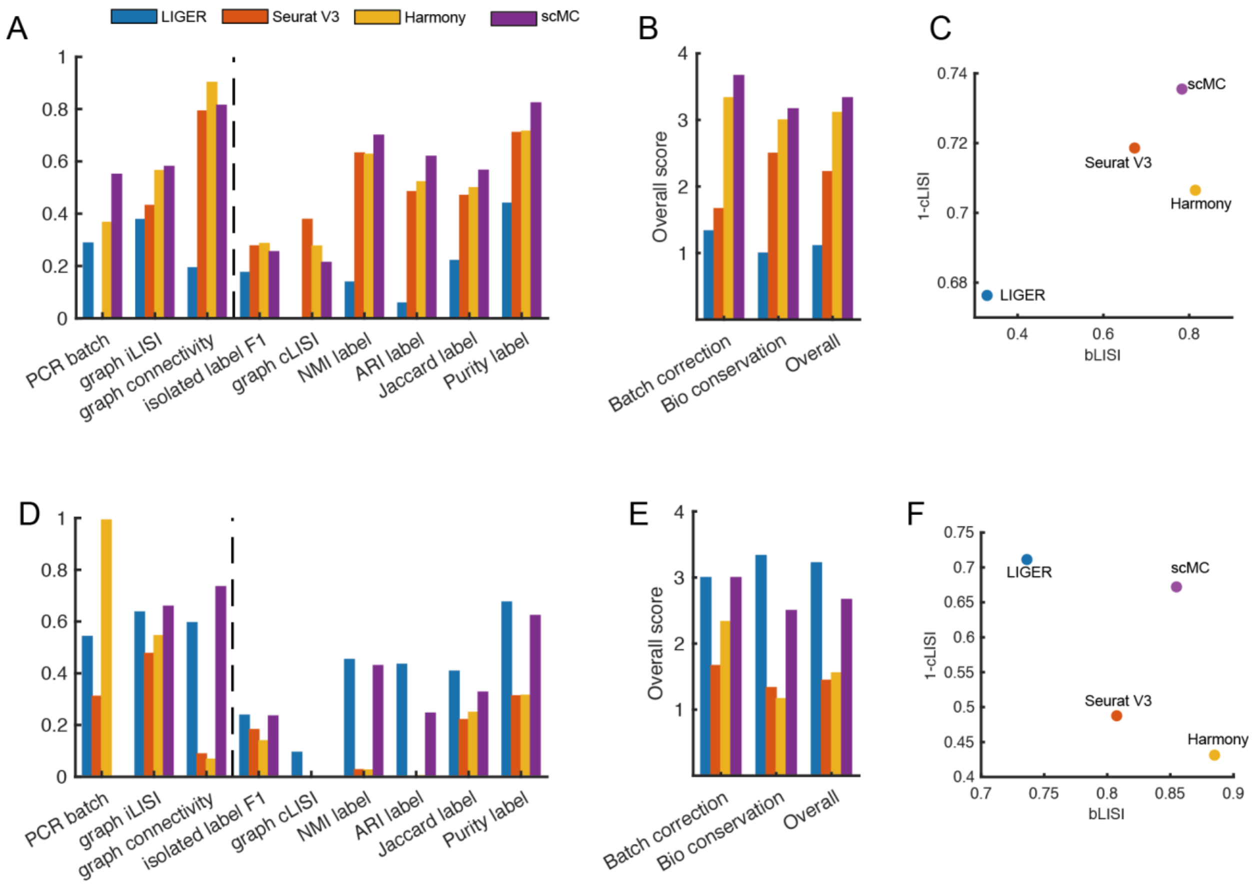

Figure S12. Comparison of integration performance using other 9 evaluation metrics on scATAC-seq dataset. (A) Evaluation of integration methods using other 9 metrics, which are grouped into two categories: batch effect removal (i..e, Batch correction) and biological variation conservation (i..e, Bio conservation) on ChromVAR-kmer transformed data. **(B)** Comparison of the overall scores among different methods, calculated based on batch effect removal metrics, biological variation conservation metrics, and both batch effect removal and biological variation conservation metrics on ChromVAR-kmer transformed data. **(C)** Dot plot showing the computed bLISI (x-axis) and 1-cLISI (y-axis) of each method on ChromVAR-kmer transformed data. The bLISI and cLISI were computed on all cells except cells in the dataset-specific clusters (i.e. Liver and Heart). One dot represents one method. scMC exhibits a good trade-off between bLISI and cLISI. **(D-F)** Comparison of integration performance on the GeneScoring transformed data.

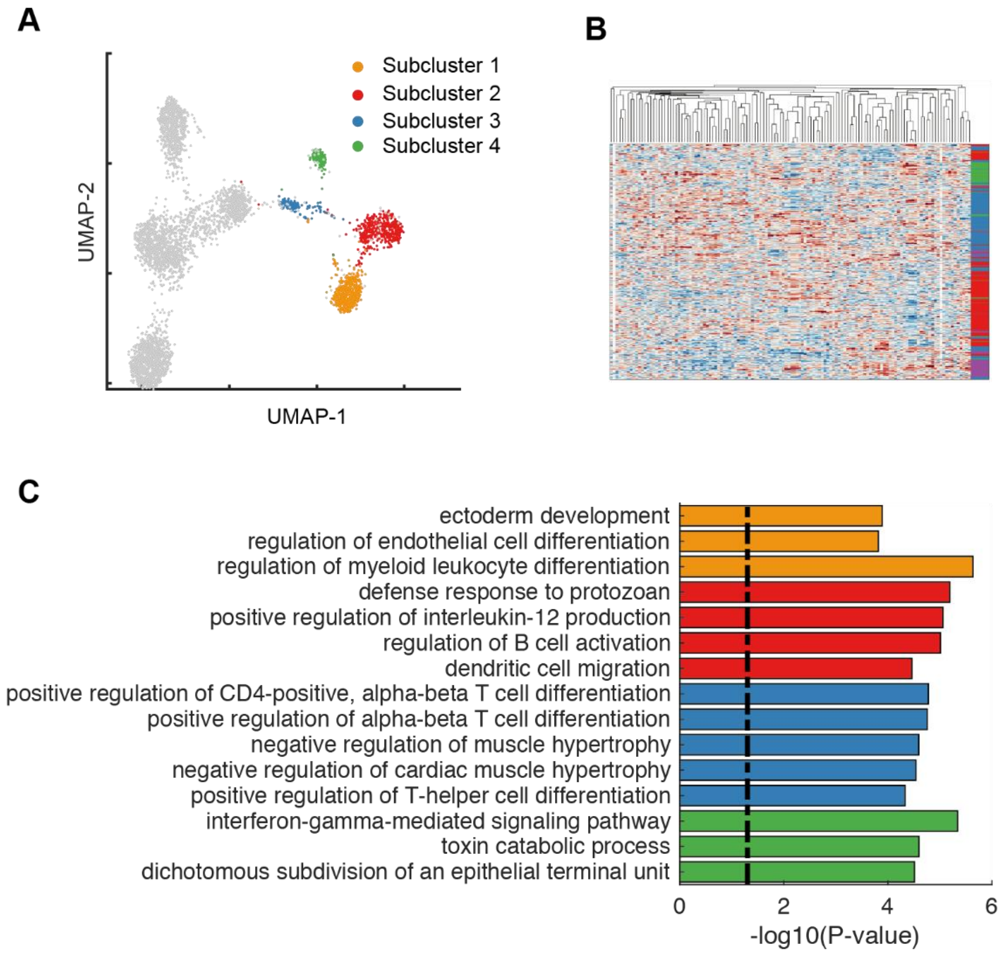

Figure S13. scMC reveals the biological heterogeneity of brain tissue on the scATAC-seq dataset. (A) UMAP visualization of the corrected data from scMC on the scATAC-seq dataset. The identified cell subpopulations in the brain tissue are highlighted and colored. Cells from other tissues are colored in grey. **(B)** Hierarchical clustering of chromVAR deviations for all the identified TFs (columns) and brain cells (rows), calculated using the differential loci among these four subpopulations. Hierarchical clustering analysis shows the patterns of these TFs were almost specific to each particular cell subpopulation, as indicated by the color bar on the right representing the group information of cells. **(C)** Enriched biological processes of the differential loci associated with each cell subpopulation.

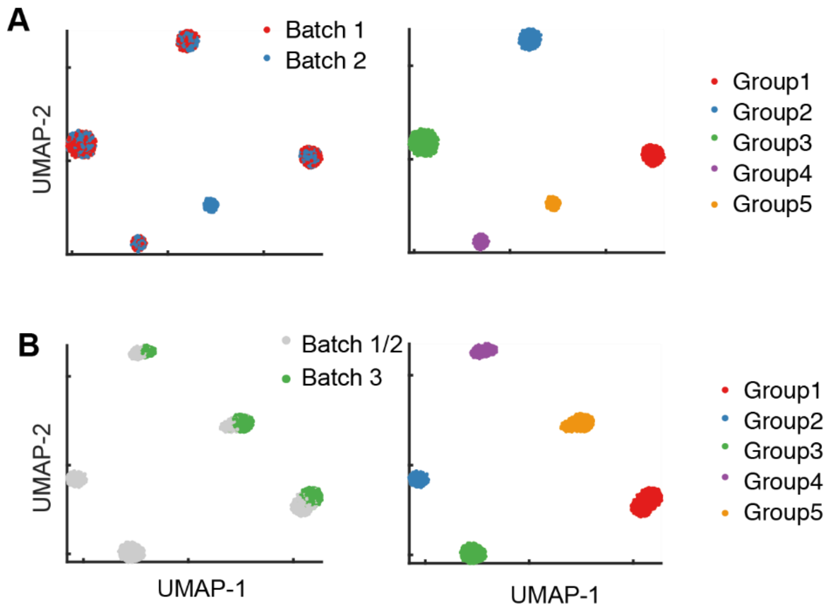

Figure S14. Transfer performance of scMC on simulated dataset 3. (A) UMAP visualization of the corrected data by applying scMC to the cells from Batch 1 and Bach 2 on simulated dataset 3. Cells are colored by batches (left) and cell types (right). **(B)** UMAP visualization of all the corrected data from Batch 1, Batch 2 and Batch 3 by projecting the cells from Batch 3 onto the correction vectors learned from Batch1 and Batch 2. Cells are colored by batches (left) and cell types (right). The cells with the same cell type labels from Batch 3 are correctly placed onto the UMAP space.

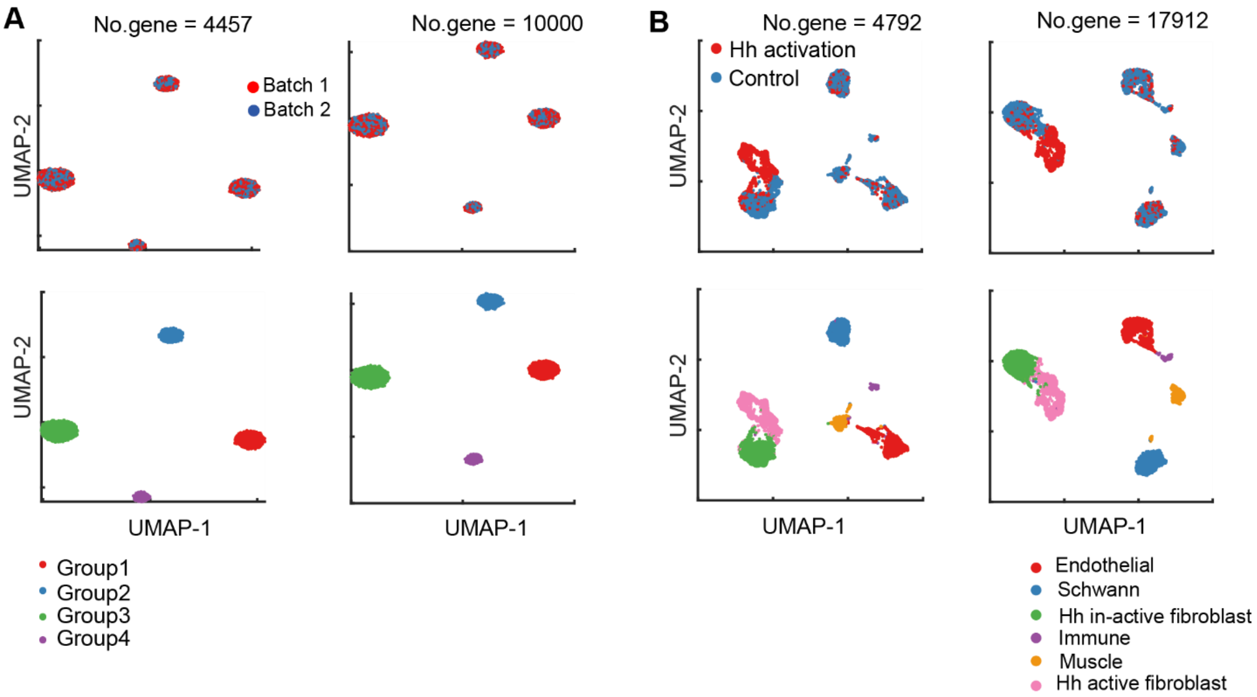

Figure S15. Performance of scMC with different number of highly variable genes (HVGs) as input. (A) UMAP visualization of the corrected data from scMC with 4457 and 10000 HVGs as input on the simulation dataset 1. Cells are colored by batches (top) and cell types (bottom). **(B)** UMAP visualization of the corrected data from scMC with 4792 and 17912 HVGs as input on the mouse skin wound healing dataset. Cells are colored by batches (top) and annotated cell labels (bottom). The Hh in-active fibroblast and Hh active fibroblast subpopulations can be consistently identified.

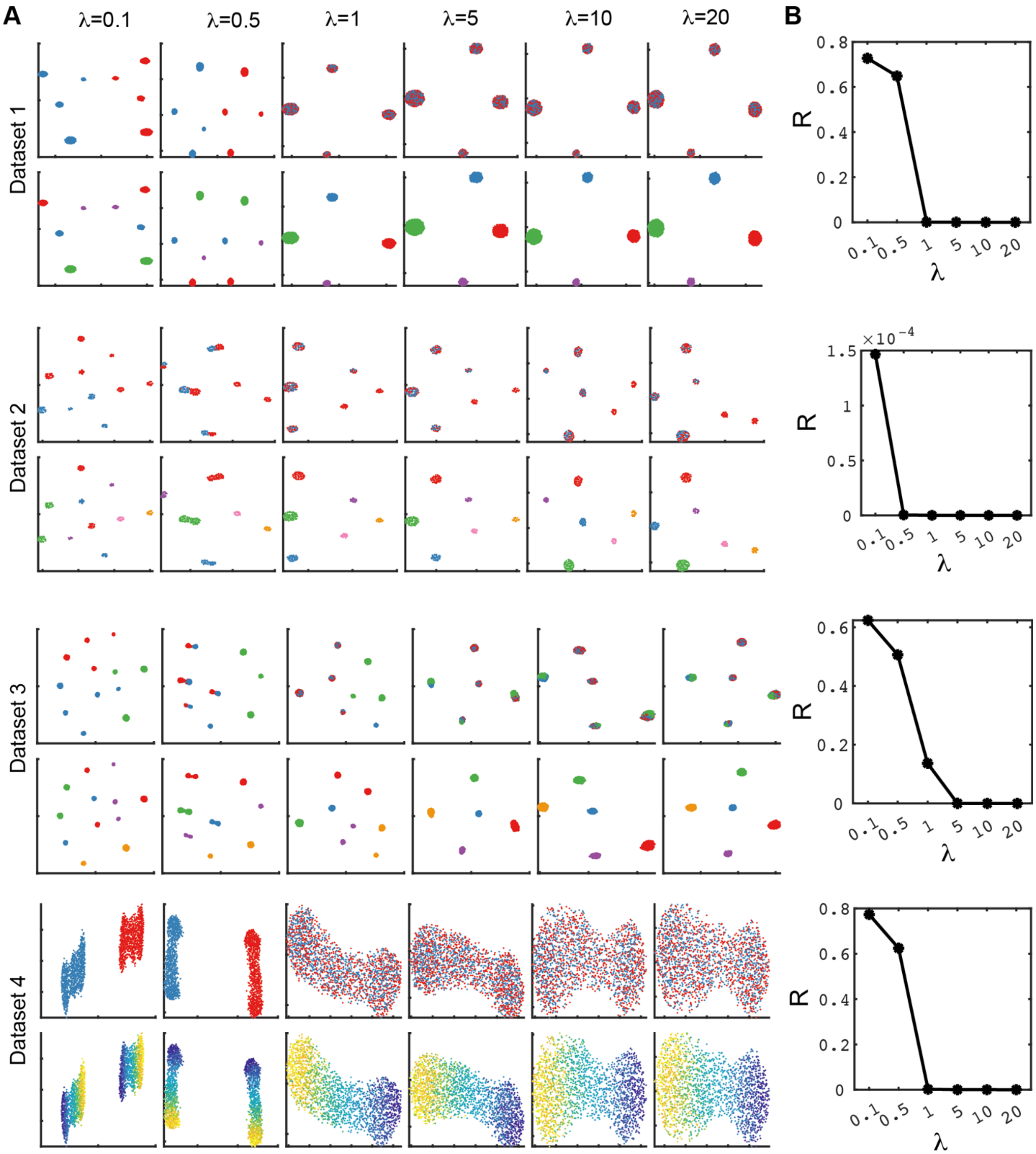

Figure S16. The performance of scMC with the varied *λ* on all simulation datasets. (A) UMAP visualization of the corrected data from scMC with *λ* varying from 0.1 to 20. For the first three datasets, cells are colored by batch labels (top row) and golden standard cell labels (bottom row). For the dataset 4, cells are colored by batch labels (top row) and golden standard cell

pseudotime values (bottom row). **(B)** The evolution of the ratio (denoted by R) of technical variation among the total variation with the increasing of λ.

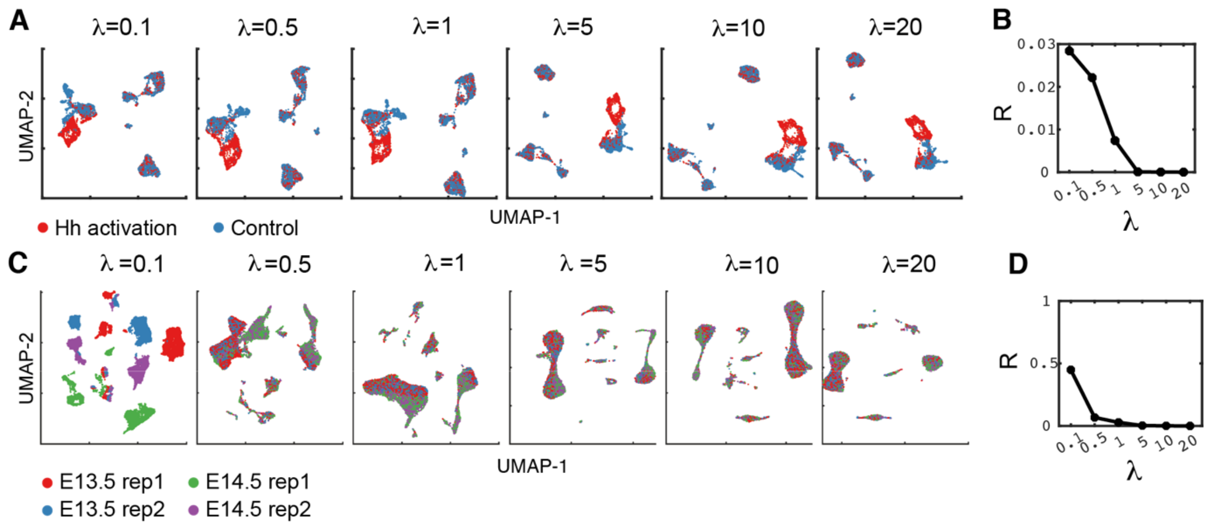

Figure S17. The performance of scMC with the varied λ on two real datasets. (A) UMAP visualization of the corrected data from scMC on the Hedgehog activation mouse skin scRNA-seq data with λ varying from 0.1 to 20. Cells are colored by experimental conditions. **(B)** The evolution of the ratio (denoted by R) of technical variation among the total variation with the increasing of λ on the control and Hedgehog activation mouse skin scRNA-seq data. **(C)** UMAP visualization of the corrected data from scMC on skin embryonic development data with λ varying from 0.1 to 20. Cells are colored by the sample identity. **(D)** The evolution of R with the increasing of λ on the skin embryonic development data.

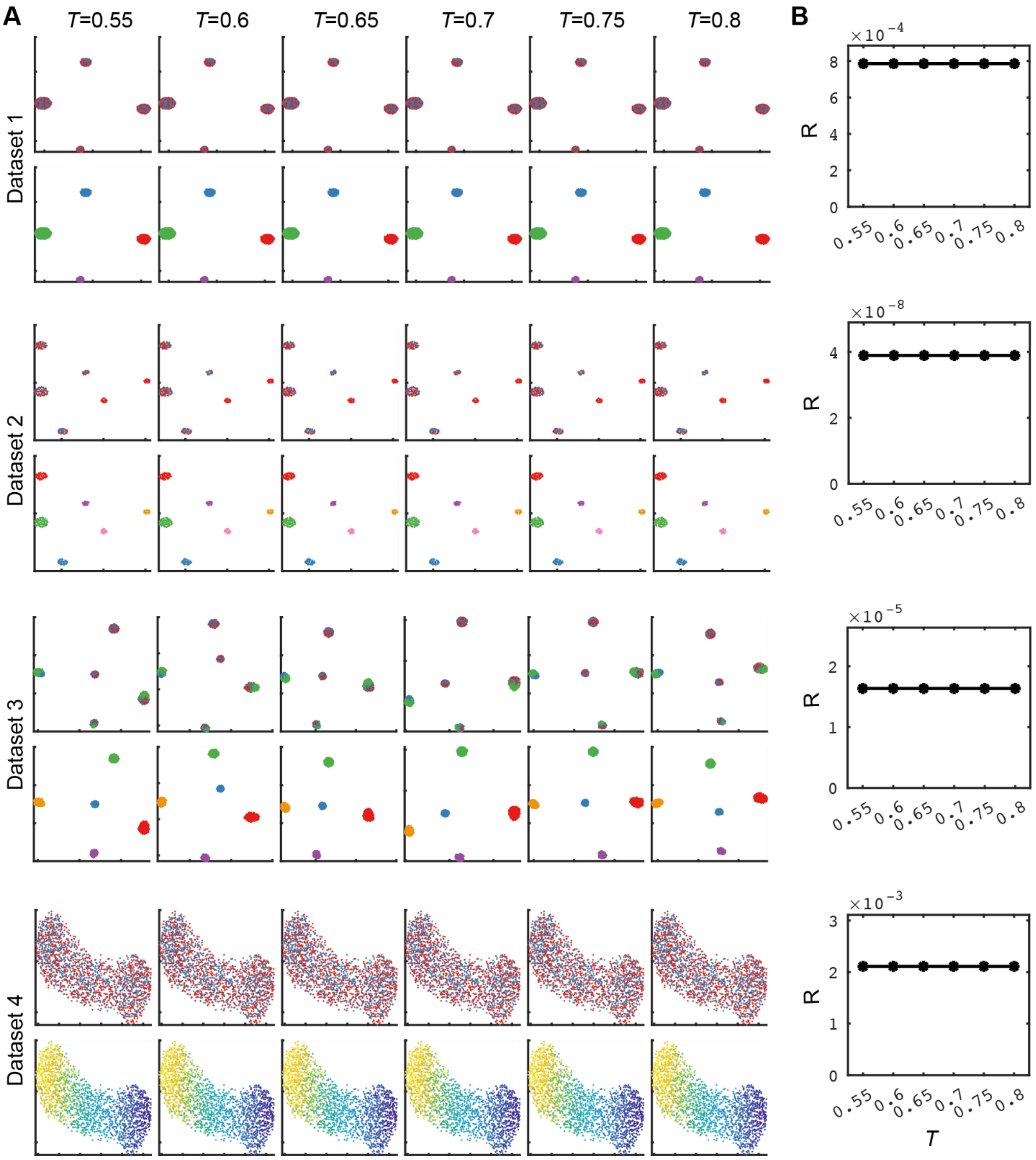

Figure S18. The performance of scMC with the varied T on all simulation datasets. (A) UMAP visualization of the corrected data from scMC with T varying from 0.55 to 0.8. For the first three datasets, cells are colored by batch labels (top row) and golden standard cell labels (bottom row). For the dataset 4, cells are colored by batch labels (top row) and golden standard cell

pseudotime values (bottom row). **(B)** The evolution of the ratio (denoted by R) of technical variation among the total variation with the increasing of *T*.

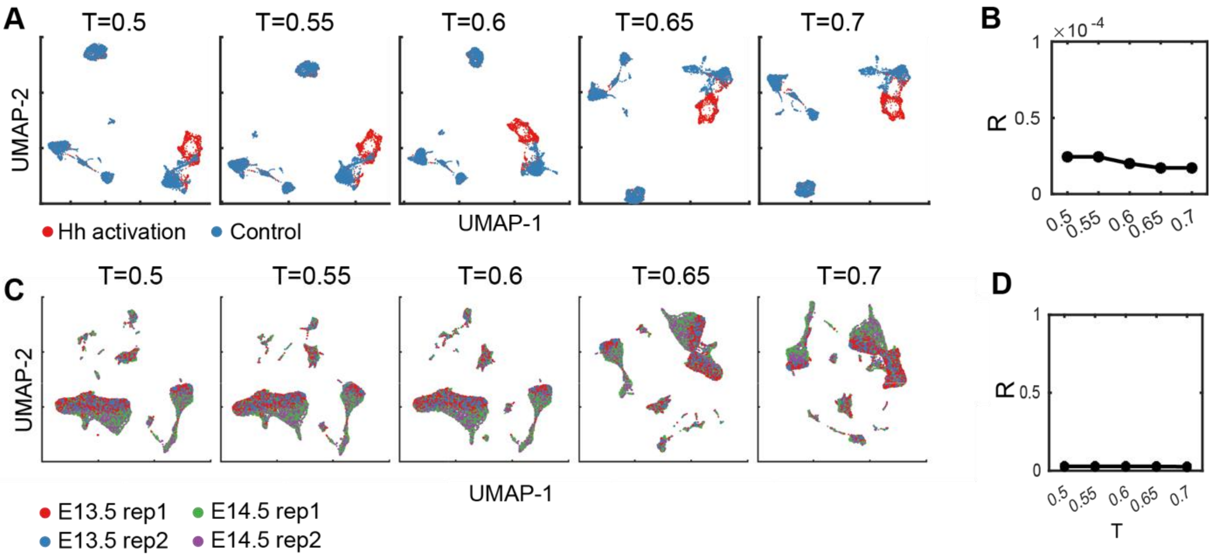

Figure S19. The performance of scMC with the varied *T* on two real datasets. (A) UMAP visualization of the corrected data from scMC on Hedgehog activation mouse skin data with *T* varying from 0.5 to 0.7. Cells are colored by experimental conditions. **(B)** The evolution of the ratio (denoted by R) of technical variation among the total variation with the increasing of *T* on the Hedgehog activation mouse skin data. **(C)** UMAP visualization of the corrected data from scMC on skin embryonic development data with *T* varying from 0.5 to 0.7. Cells are colored by the sample identity. **(D)** The evolution of *R* with the increasing of *T* on the skin embryonic development data.
