# Supplementary material for: scMC learns biological variation through the alignment of multiple single-cell genomics datasets
Source: Genome Biol. 2021 Jan 4;22:10. doi: 10.1186/s13059-020-02238-2 (PMC7784288; doi:10.1186/s13059-020-02238-2)
Supplement: Supplementary file 3 — Additional file 3. Review history. [file 13059_2020_2238_MOESM3_ESM.docx]

Review History

**First round of review**

**Reviewer 1**

**Were you able to assess all statistics in the manuscript, including the appropriateness of statistical tests used?**

Yes

**Were you able to directly test the methods?**

No

**Comments to author:**

Review of "scMC learns biological variation through the alignment of multiple single cell genomics datasets" (GBIO-D-20-01063)

\# Summary

The authors present scMC, a data integration method that uses the leiden clustering algorithm to group cells in the separate batches and aligns shared cell clusters based on similarity scores. The approach resembles the mutual nearest neighbors (MNN) algorithm, but the authors compute a technical confounder matrix in addition to distinguish between biological and technical variation. The approach was shown to separate different cell types and loosely merges the same cell types from different conditions. Furthermore, the applicability and performance of scML was shown to preserve developmental trajectories and to be able to integrate scATACseq data in a meaningful way. scML outperformed models such as Seurat v3, Harmony and LIGER in many settings, but not in all examples. The novelty of scML is that it integrates well datasets with differences in cell type composition and scATACseq-derived feature matrix datasets.

However, the manuscripts shows several weaknesses in not so much approach but benchmarking that the authors IMHO should address to really demonstrate novelty and impact.

\# Major comments

* The authors state in the introduction that "However, these methods did not explicitly distinguish technical variation from biological variation when aligning multiple single cell datasets, which might mitigate the biological variation as well when removing the technical variation." In their examples, they integrate two batches (Fig. 3,4) of single-cell RNAseq data. In their simulation study, only one out of four scenarios contained three batches, the others contained only two. Therefore, the single cell RNAseq data examples lack complexity. Luecken et al (biorxiv, 2020) have shown that the top performing methods performed less when used to integrate multiple batches. Hence, the authors should follow their own initial motivation to integrate multiple datasets and show a single-cell RNAseq integration example with more than 2 batches to integrate. Further, the authors should show how scML deals with an increasing number of batches in a simulation study.

* lines 45-47 (page 2): I'd recommend the authors should to include recent larger-scale benchmarking from Chazarra-Gil et al (biorxiv 2020) and Luecken et al to set their study into context, esp. regarding their point on alignment of multiple datasets, which was not covered in Tran et al (ref 17).

* In general, given the above cited many recent comparisons for batch integration methods, I strongly recommend the authors to implement or just use the indices from these studies as well as those examples to benchmark. The benchmarks come with tools for comparisons, and I think readers would really like to see comparison on exactly those to judge quality.

* The inconsistent choice of metrics to quantify batch effect removal was confusing. Simulated data: metrics for evaluation were NMI, ARI, Jaccard index and Purity. IN later examples, the authors used silhouette scores, an F1-score version of the LISI scores and a so-called specificity score. I wonder why no single cell specific measures were used on the simulated data? Vice versa, why did they not apply the NMI, ARI, Jaccard Index and Purity on the real data? I fail to see the motivation to use different metrics on different datasets.

* P.6 line 50: incorrect reference. The authors introduce the LISI score with ref. 13 (scMerge publication), but the correct one would be Korsunsky et al (ref 10). Further, the authors have to clarify and explain that they are not using the original formulation of the LISI score, but a derived F1-score (see methods), that assesses both batch mixing and cell type separation.

* In the case of embryonic skin development (Fig. 5, 4 batches from 2 timepoints), it was not clear whether the authors used a two-step approach to integrate the data of if they integrated all 4 batches at once - from the text, I have got the impression that the authors applied a two-step integration (first replicates of the same time point, then integration across time points). Such a procedure is unwise as it tends to overcorrect the data. As far as I am aware, models like Harmony are able to deal with this type of nested batch effects directly.

* The authors do not compare the recovered trajectories in a quantitative fashion. Clearly, the visual inspection is not enough and I fail to see the differences of the PHATE plots of scMC and Harmony or LIGER, esp. for the epidermal trajectory. Saelens et al. (Nat Biotech, 2019) gives an excellent overview on the comparison of pseudotime algorithms and thus reconstructed trajectories.

* Discussion: The discussion does not earn the name discussion. It is rather an extensive summary of results, and discussion of parameters and runtime. The parameter discussion should be done as a first section in the results part OR moved to the supplement. Runtime comparison is definitely a result and should be presented as such, not as part of the discussion. Further, I would like to see an argument about the relation of batch effects and biological signals. The authors seem to assume that both sources of variation can be clearly separated and they should elucidate why scMC is capable to that while others fail at that task. Moreover, I think that discussing the reconstruction of pseudotime trajectories would improve the quality of the discussion and the manuscript.

* Conclusions: The conclusions read like a throwaway statement. I cannot follow the statement of "deepen our understanding how distinct cells respond to perturbation or disease." scMC is a data integration method after all and cannot be used to predict perturbations, in contrast to e.g. scGEN (Lotfollahi et al., Nat Meth, 2019).

\# Minor comments

* Incorrect reference of the LISI score (Method section): In the methods section about the LISI score, the authors cite Buttner et al. (ref 37) as reference for the LISI score, but the correct citation should be Korsunsky et al (ref 10), further, the F1-score like LISI score reference is again the scMerge publication (ref 13) and I fail to see why the authors referenced it, because the LISI score was not used in the referenced publication. Overall, I suggest that the authors thoroughly go over their references and check if they give credit to the publications they intended to.

* In the UMAP plots, it would definitely help to randomize the plotting order of the points, because when the cells from one batch are plotted on top of the other, it is impossible to inspect the cell distribution by eye.

* In fig. 2a, a legend of the cell types (lower panel) is missing and makes it harder to understand the display.

* P.5 line 54 - golden standard? I would write "gold standard"

* Line numbers do not match with text lines and it's hard to assign a text line to a line number. Further, the numbers start over on every page.

* Dataset description: The authors do not describe how many batches are present in each of the tested scenarios

* Fig 4b: The grey circle in Seurat UMAP is not described in the figure legend. Further, the colors of the cell types seem to change over the different plots and it is impossible to follow the authors's argument on fibroblast subpopulations. The cell type assignment needs to be consistent over all panels and a common legend for both batches and cell types would definitely help to understand the authors's statements.

* Fig. 6: Please reconsider the choice of color palette as the colors are not particularly colorblind-friendly.

* P. 17: The link (http://htmlpreview.github.io/?https://github.com/immunogenomics/harmony/blob/master/docs/SeuratV3.html) is broken.

* "scMC showed the largest silhouette index value than other three methods (Fig. 6b)" - grammar: "compared to" instead of "than"

**Reviewer 2**

**Were you able to assess all statistics in the manuscript, including the appropriateness of statistical tests used?**

Yes: fine

**Were you able to directly test the methods?**

No

**Comments to author:**

The paper focuses on an important, though extensively studied problem: Correction of batch or experiment effects in scRNA-Seq and ATAC-Seq data. The key novelty in this paper is the attempt to distinguish between biological noise (i.e. variation that is of interest) and technical / experimental noise. This is achieved by learning correction terms based on a stringent matching of a subset of the cells (cluster specific cells) and using these for the rest of the cells.

The method, termed scMC is applied to several simulated and real datasets and compared to three state of the art methods that have previously been shown to achieve good results in alignment studies. Results indicate that scMC can improve on these other methods, at least for some of the real datasets. While Results seem to be strong, and the method seems to successfully identify batch related impacts, there are a number of issues with the description of the method that are not clear. These and some clarification of the results need to be improved before the paper can be accepted.

Major comments:

1. Several aspects of the Method description are not clear.

a. What if a cluster in one dataset does not match any cluster in another dataset? Since you are using a match cutoff, if no cluster in the other dataset is matched how are the cells in this cluster corrected?

b. I was surprised that the correction terms are cluster specific. Why is that? Why not learn global variance correction terms? This way you can correct for all cells, whether they are in a matched cluster or not. This would also allow you to correct for additional data from the same lab when it becomes available rather than re-running the method from scratch.

c. How many genes were selected as features for each of the datasets you analyzed? Did you try other thresholds for selecting such features? Since correction is only learned for selected genes, if they are not included there will be no way to study them in the joint space.

d. Can you explain the following sentence 'The number of putative cell clusters is determined by the number of singular values associated with the consensus matrix at which the increase ratio between adjacent singular values is smaller than 0.01.'? How many clusters do you have for each of the datasets? Are some cell types divided between multiple clusters or do you usually see a single cluster for each type?

e. Selection of marker genes for cluster matching: Is this done only on the confident cells or using all cells in the cluster? If the latter, why? Since only the confident ones are used to learn the correction why not use them to determine the matching as well?

f. Selection of marker genes: What likelihood ratio tests are you referring to here? One vs. all? What exactly is being compared to identify these markers?

g. Matching clusters: Can you have many to many matchings? If so, how do you handle the multiple correction factors you learn for the same class?

h. In the section: 'Learning the correction vectors', line 50 on page 16. I don't understand the new optimization function for V. Isn't it fixed already by the assignment from the above optimization function (top of page 16)?

i. (side comment - would have been very useful to number of the equations). Line 20 on page 16. This seems to be sensitive to the ordering of the cells, no? Why is this correct? Why not have a function that is independent of the ordering?

j. What do you mean by 'randomly subset' (line 11 on that page)? Do you mean that you remove a subset of the cells? Why? How is this affecting the results? Can you better explain that equation?

k. LISI calculation - Why are you presenting the average LISI and not the two components separately? Basically cLISI is the same as specificity, why do you need both? Replace the LISI result with b and c LISI and remove specificity since its redundant with cLISI.

2. The paper is very light on any biological insights that the new alignment provides. While this is a methods paper it would be good to discuss this a bit more, especially for the ATAC-Seq data

3. Can you explicitly mention the run time for different sized (not just point to the supplement) and also mention if the run time is linearly or quadratically or any other complexity term related to the size of the input? Would the method work on 100K cells? 1M cells?

4. There are several typos in the paper and it would greatly benefit from English editing. A few examples:

' Due to rapid technological development, the single cell datasets have been generated under different' -> 'Due to rapid technological development, single cell datasets have been generated under different'

'both Seurat V3 and scMC well removed batch effects' -> 'both Seurat V3 and scMC successfully removed batch effects' (several places)

'We then respectively reconstructed the epidermal and dermal trajectories' -> 'We then reconstructed the epidermal and dermal trajectories respectively'

**Authors Response**

**Point-by-point responses to the reviewers’ comments:**

Reviewer reports

Reviewer #1

\# Summary

The authors present scMC, a data integration method that uses the leiden clustering algorithm to group cells in the separate batches and aligns shared cell clusters based on similarity scores. The approach resembles the mutual nearest neighbors (MNN) algorithm, but the authors compute a technical confounder matrix in addition to distinguish between biological and technical variation. The approach was shown to separate different cell types and loosely merges the same cell types from different conditions. Furthermore, the applicability and performance of scML was shown to preserve developmental trajectories and to be able to integrate scATACseq data in a meaningful way. scML outperformed models such as Seurat v3, Harmony and LIGER in many settings, but not in all examples. The novelty of scML is that it integrates well datasets with differences in cell type composition and scATACseq-derived feature matrix datasets.

However, the manuscript shows several weaknesses in not so much approach but benchmarking that the authors IMHO should address to really demonstrate novelty and impact.

*Response: We thank the reviewer very much for the appreciation of scMC’s novelty and the insightful comments. Below please find our detailed responses to the reviewer’s comments. Multiple changes were introduced throughout the manuscript and all changes were highlighted in red in the revised manuscript, including both main text and Additional files.*

\# Major comments

* The authors state in the introduction that "However, these methods did not explicitly distinguish technical variation from biological variation when aligning multiple single cell datasets, which might mitigate the biological variation as well when removing the technical variation." In their examples, they integrate two batches (Fig. 3,4) of single-cell RNAseq data. In their simulation study, only one out of four scenarios contained three batches, the others contained only two. Therefore, the single cell RNAseq data examples lack complexity. Luecken et al (biorxiv, 2020) have shown that the top performing methods performed less when used to integrate multiple batches. Hence, the authors should follow their own initial motivation to integrate multiple datasets and show a single-cell RNAseq integration example with more than 2 batches to integrate. Further, the authors should show how scML deals with an increasing number of batches in a simulation study.

*Response: Thanks for the nice suggestions. In the original manuscript, in addition to the simulation dataset with three batches, the mouse embryonic development scRNA-seq dataset we analyzed actually consists of four batches including two time points and two samples per each time point (Figure 5). We have made this point clearer in the Results and Methods sections in the revised manuscript. Moreover, we agree with the reviewer that these datasets lack complexity, especially comparing with the datasets used in Luecken et al (biorxiv 2020).*

*To address this issue, in the revised manuscript we have tested scMC on two simulation datasets and one mouse lung scRNA-seq dataset used in the benchmarking paper (Luecken et al, biorxiv 2020). These two simulation datasets were marked as dataset 5 and dataset 6. Dataset 5 consists of 12,097 cells with six batches and seven cell groups, and dataset 6 consists of 19,318 cells with 16 nested sub-batches (four sets of four sub-batches) and 4 cell groups. By visually scrutinizing the projected cells in the integrated UMAP space, on simulation dataset 5, all integration methods including LIGER, Seurat V3, Harmony and scMC could remove batch effects and detect the true cell groups. However, scMC exhibits better performance in preserving cell identity labels than other methods. LIGER, Seurat V3 and Harmony clearly misplaced some cells in the integrated UMAP space (the fifth row of updated Figure 2a).*

*Moreover, in the light of the reviewer’s suggestion, in the revision we have evaluated these methods using another 9 metrics proposed in Luecken et al, biorxiv, 2020. Five metrics were used to assess batch removal, including PCR batch, batch ASW, graph iLISI, kBET and graph connectivity, and four metrics were used to assess conservation of biological variation, including label ASW, isolated label F1, isolated label silhouette, graph cLISI. We also evaluated these methods using other metrics that were used in our original manuscript. In sum, we computed 16 evaluation metrics for each method. We defined overall scores by averaging the rank of each method based on three categories of metrics, including batch effect removal metrics, biological conservation metrics and both batch effect removal and biological conservation metrics. A larger overall score indicates better performance. On simulation dataset 5, comparing to LIGER, Seurat V3 and Harmony, scMC consistently showed higher biological conservation scores using various metrics (the fifth row of updated Figure 2b), and had the highest overall scores in both batch effect removal and biological conservation (the fifth row of updated Figure 2c).*

*On simulation dataset 6 with 16 nested sub-batches and 4 cell groups, LIGER and Seurat V3 removed batch effects but showed a complete mixture of cell subpopulations (the sixth row of Fig. 2a), leading to higher batch removal scores and lower biological conservation scores (the sixth row of Figs 2b and 2c). Harmony partially merged different batches and retained biological variation with some mixed cell labels (the sixth row of Fig. 2a). Nevertheless, scMC could retain cell identify labels while removing batch effects, leading to moderate batch removal scores and consistently higher biological conservation scores. Based on the overall scores, scMC shows the highest scores in both batch effect removal and biological conservations (the sixth row of Fig. 2c). We noted different performance of Seurat V3 and Harmony in comparison with the results in Luecken et al.’s recent work (biorxiv 2020) on this simulation dataset 6, which might be caused by the use of different methods for selecting highly variable genes (HVGs). In particular, Luecken et al. run Seurat V3 and Harmony using HVGs from Scanpy, while we run them based on the standard integration workflow provided online, i.e., using the ‘FindVariableFeatures’ function from Seurat package. Such differences suggest the importance of HVGs for data integration. We provided a reproducible benchmarking codes for both simulation datasets 5 and 6 in Github (https://github.com/amsszlh/scMC/tree/master/benchmarking). In the revised manuscript, we have added these analyses in the Results section (Page 6).*

*Furthermore, we have applied scMC to a mouse lung scRNA-seq dataset that was kindly provided by MD Luecken and downloaded from https://figshare.com. This lung dataset contains 32,472 cells across 16 batches. The batch labels and annotated cell labels were used for computing evaluation metrics. All the tested methods performed well in batch effect removal. In particular, Seurat V3 had the highest overall batch removal score while scMC exhibited a moderate batch removal score in comparison with other methods (new Figs. 6a-c). However, Seurat V3 performed poorly in preserving the biological variation, making the various cell subpopulations more compact in the UMAP space along with some mixed cell labels and leading to lower biological conservation scores. Seurat V3 significantly reduced the biological variation among different cell types, such as epithelial cells (i.e., ciliated cells, basal 1 and basal 2), fibroblasts and immune cells (i.e., B cells, endothelial cells and mast cells). Nevertheless, LIGER, Harmony and scMC succeeded in separating these broad cell types. Compared to scMC with the highest biological conservation scores, LIGER separated the ciliated cells into different groups and were not able to discriminate basal 1 from basal 2 cells and neutrophils from macrophage, leading to the lowest biological conservation scores, in particular for ASW label and ARI label. Harmony exhibited good separation of different cell types, but showed an overlap between neutrophils, dendritic cells and macrophage, leading to relatively lower biological conservation scores compared to scMC. scMC can not only separate the different broad cell types, but also discriminate the biologically similar cell states, such as basal 1 and basal 2, macrophage and dendritic cells. Quantitatively, scMC showed moderate batch removal scores, however, the highest biological conservation scores as well as the overall score (new Fig. 6c). These new results further demonstrate the better performance of scMC in retraining biological variation while removing batch effects. We also provided a reproducible benchmarking codes for this mouse lung dataset in Github (https://github.com/amsszlh/scMC/tree/master/benchmarking). In the revised manuscript, we have added these analyses in the Results section (Page 12).*

* lines 45-47 (page 2): I'd recommend the authors should to include recent larger-scale benchmarking from Chazarra-Gil et al (biorxiv 2020) and Luecken et al to set their study into context, esp. regarding their point on alignment of multiple datasets, which was not covered in Tran et al (ref 17).

*Response: We would like to thank the reviewer for raising this important point. In the revised manuscript, we have added the following sentence in Introduction section.*

*“However, recent large-scale benchmarking studies (Chazarra-Gil et al, bioRxiv, 2020; Luecken et al, bioRxiv 2020) suggested that method performance is dependent on the complexity of the integration task and multiple batches introduce additional difficulties for those methods that perform well for two batches.”*

* In general, given the above cited many recent comparisons for batch integration methods, I strongly recommend the authors to implement or just use the indices from these studies as well as those examples to benchmark. The benchmarks come with tools for comparisons, and I think readers would really like to see comparison on exactly those to judge quality.

*Response: We thank the reviewer very much for pointing out these two important and timely benchmarking studies and providing the insightful suggestions. As mentioned above, we have evaluated scMC on two simulation datasets and one mouse lung scRNA-seq dataset using additional 9 evaluation metrics that were used in Luecken et al, biorxiv 2020. These 9 evaluation metrics were computed using the scIB.metrics.metrics() function in the excellent tool scIB (https://github.com/theislab/scib). In addition, we computed these metrics on other datasets used in our manuscript, and updated the related figures (updated Figure 2, and new Additional file 2 Figure S16 related to Figure 7). These new results further demonstrate the better performance of scMC in retraining biological variation while removing batch effects.*

* The inconsistent choice of metrics to quantify batch effect removal was confusing. Simulated data: metrics for evaluation were NMI, ARI, Jaccard index and Purity. IN later examples, the authors used silhouette scores, an F1-score version of the LISI scores and a so-called specificity score. I wonder why no single cell specific measures were used on the simulated data? Vice versa, why did they not apply the NMI, ARI, Jaccard Index and Purity on the real data? I fail to see the motivation to use different metrics on different datasets.

*Response: We apologize for not being more careful in choosing the metrics. In the revised manuscript we have added the Silhouette scores, F1-sore version of the LISI scores (denoted by LISI-derived F1 score) and the Specificity score to the simulation datasets (updated Figure 2). We also applied both NMI, ARI, Jaccard and Purity scores to other datasets whenever the gold standard label information is available (new Additional file 2 Figure S16).*

* P.6 line 50: incorrect reference. The authors introduce the LISI score with ref. 13 (scMerge publication), but the correct one would be Korsunsky et al (ref 10). Further, the authors have to clarify and explain that they are not using the original formulation of the LISI score, but a derived F1-score (see methods), that assesses both batch mixing and cell type separation.

*Response: Thanks for raising this point. We have now corrected the references and clarified the differences between the original LISI score and the derived F1-score in this revision (Page 26). We have also updated our figures to use the term “LISI-derived F1-score” instead of “LISI”. In sum, to assess both batch mixing and cell type separation that are assessed by bLISI and cLISI, we summarized these two metrics into a single F1 score using a similar formular in a previous study (Lin et al, PNAS 2019). We term this new score as LISI-derived F1 score.*

* In the case of embryonic skin development (Fig. 5, 4 batches from 2 timepoints), it was not clear whether the authors used a two-step approach to integrate the data of if they integrated all 4 batches at once - from the text, I have got the impression that the authors applied a two-step integration (first replicates of the same time point, then integration across time points). Such a procedure is unwise as it tends to overcorrect the data. As far as I am aware, models like Harmony are able to deal with this type of nested batch effects directly.

*Response: In the embryonic skin development dataset, we performed two integration tasks: integrating the replicate samples of each time point individually, and integrating all 4 batches at once. In the revised manuscript we have clarified this point (Page 10).*

* The authors do not compare the recovered trajectories in a quantitative fashion. Clearly, the visual inspection is not enough and I fail to see the differences of the PHATE plots of scMC and Harmony or LIGER, esp. for the epidermal trajectory. Saelens et al. (Nat Biotech, 2019) gives an excellent overview on the comparison of pseudotime algorithms and thus reconstructed trajectories.

*Response: We thank the reviewer for this great suggestion. Using a similar method in Luecken et al., biorxiv 2020 that assumed that trajectories found in the unintegrated data for each batch gave the most accurate biological signal, we have quantitatively evaluated the recovered trajectories by computing Pearson correlation coefficients between the pseudotime values before and after integration. To infer the pseudotime values of cells, we adopt a principal curve-based approach used in the previous pseudotime-inference tools such as scEpath (Jin et al, Bioinformatics 2018) and Slingshot (Street et al, BMC Genomics 2018). Specifically, we fitted a principal curve in the PHATE space and obtain the pseudotime values by orthogonal projections of cells onto the curves. Correlation analysis were carried out for the pseudotime values of cells on the trajectory before and after integration. Consistent with our visual observation, LIGER, Harmony and scMC performed well in preserving cell pseudotime for the epidermal trajectory, in particular for the cells from E14.5 at which cells can progress toward the suprabasal cell state (updated Fig. 5h). For the dermal trajectory, although Harmony did not produce a clear trajectory in the PHATE space (Additional file 2 Figure S8f), it showed comparable correlation values with scMC (updated Fig. 5h). However, LIGER and Seurat V3 produced lower correlation values for the dermal trajectory due to the loss of trajectory structure. In addition, we investigated the pseudotemporal dynamics of several marker genes based on the scMC-derived integrated developmental trajectories. As expected, during epidermal and dermal differentiation, the temporal dynamics of undifferentiated (e.g., Krt14 and Lum) and differentiated (e.g., Krt10, Lor, Sox2 and Bmp4) markers were downregulated and upregulated over pseudotime (updated Fig. 5j). scMC correctly captures the development process using datasets from different time points and replicates, showing meaningful gene dynamics. In the revised manuscript, we updated Figure 5 to add these new results.*

* Discussion: The discussion does not earn the name discussion. It is rather an extensive summary of results, and discussion of parameters and runtime. The parameter discussion should be done as a first section in the results part OR moved to the supplement. Runtime comparison is definitely a result and should be presented as such, not as part of the discussion. Further, I would like to see an argument about the relation of batch effects and biological signals. The authors seem to assume that both sources of variation can be clearly separated and they should elucidate why scMC is capable to that while others fail at that task. Moreover, I think that discussing the reconstruction of pseudotime trajectories would improve the quality of the discussion and the manuscript.

*Response: Thanks for the nice suggestion. In the revised manuscript we moved the parameter discussion to the Additional file 1, and the runtime comparison to the Results section (Page 7). We have re-written the entire Discussion section (Pages 15-18).*

* Conclusions: The conclusions read like a throwaway statement. I cannot follow the statement of "deepen our understanding how distinct cells respond to perturbation or disease." scMC is a data integration method after all and cannot be used to predict perturbations, in contrast to e.g. scGEN (Lotfollahi et al., Nat Meth, 2019).

*Response: Thanks for pointing it out. We modified our statement in Conclusion section. It now reads as “The capability of scMC in preserving biological signals while removing batch effects will facilitate biological discoveries from the ever-growing number of single cell studies.”.*

\# Minor comments

* Incorrect reference of the LISI score (Method section): In the methods section about the LISI score, the authors cite Buttner et al. (ref 37) as reference for the LISI score, but the correct citation should be Korsunsky et al (ref 10), further, the F1-score like LISI score reference is again the scMerge publication (ref 13) and I fail to see why the authors referenced it, because the LISI score was not used in the referenced publication. Overall, I suggest that the authors thoroughly go over their references and check if they give credit to the publications they intended to.

*Response: We thank the reviewer for the careful reading of our manuscript. In the revised manuscript, we have carefully checked our references and corrected them.*

* In the UMAP plots, it would definitely help to randomize the plotting order of the points, because when the cells from one batch are plotted on top of the other, it is impossible to inspect the cell distribution by eye.

*Response: Thanks for the nice suggestion. In the revised manuscript we have re-generated all the UMAP plots in the figures by randomizing the plotting order of points.*

* In fig. 2a, a legend of the cell types (lower panel) is missing and makes it harder to understand the display.

*Response: In the revised manuscript we have added a legend of the cell types.*

* P.5 line 54 - golden standard? I would write "gold standard"

*Response: In the revised manuscript, we replaced ‘golden standard’ by ‘gold standard’.*

* Line numbers do not match with text lines and it's hard to assign a text line to a line number. Further, the numbers start over on every page.

*Response: The line numbers were automatically added by the online submission system.*

* Dataset description: The authors do not describe how many batches are present in each of the tested scenarios

*Response: In the revised manuscript we have added the detailed information in the section of Availability of Data and Materials.*

* Fig 4b: The grey circle in Seurat UMAP is not described in the figure legend. Further, the colors of the cell types seem to change over the different plots and it is impossible to follow the authors's argument on fibroblast subpopulations. The cell type assignment needs to be consistent over all panels and a common legend for both batches and cell types would definitely help to understand the authors's statements.

*Response: Due to the lack of ground truth, we carefully annotated the cell clusters identified by scMC based on the expression patterns of known markers (Additional File 2 Figure S3). In the revised manuscript, the cell type assignment was colored in all methods using our manual annotations. The grey circle in the Seurat UMAP plot indicates the fibroblast cells. In the revised manuscript, we have removed the grey circle since a consistent cell type legend was used now.*

* Fig. 6: Please reconsider the choice of color palette as the colors are not particularly colorblind-friendly.

*Response: We have used colorblind-friendly colors in the updated Fig. 7 (the Fig. 6 in the original manuscript) in the revised manuscript.*

* P. 17: The link (http://htmlpreview.github.io/?https://github.com/immunogenomics/harmony/blob/master/docs/SeuratV3.html) is broken.

*Response: We have re-checked this link and found it works well. We run Harmony using the vignette named “Seurat V3” provided in https://github.com/immunogenomics/harmony.*

* "scMC showed the largest silhouette index value than other three methods (Fig. 6b)" - grammar: "compared to" instead of "than".

*Response: We have corrected it in the revised manuscript.*

Reviewer #2

Reviewer #2: The paper focuses on an important, though extensively studied problem: Correction of batch or experiment effects in scRNA-Seq and ATAC-Seq data. The key novelty in this paper is the attempt to distinguish between biological noise (i.e. variation that is of interest) and technical / experimental noise. This is achieved by learning correction terms based on a stringent matching of a subset of the cells (cluster specific cells) and using these for the rest of the cells.

The method, termed scMC is applied to several simulated and real datasets and compared to three state of the art methods that have previously been shown to achieve good results in alignment studies. Results indicate that scMC can improve on these other methods, at least for some of the real datasets. While Results seem to be strong, and the method seems to successfully identify batch related impacts, there are a number of issues with the description of the method that are not clear. These and some clarification of the results need to be improved before the paper can be accepted.

*Response: We thank the reviewer very much for the appreciation of the importance and novelty of our work and the insightful comments. Below please find our detailed responses to the reviewer’s comments. All changes were highlighted in red in the revised manuscript, including both main text and Additional files.*

Major comments:

1. Several aspects of the Method description are not clear.

*Response: We thank the reviewer for the careful reading of our manuscript and insightful comments. We have clarified many details in the Method section in the revised manuscript. Please see below for details on this point.*

a. What if a cluster in one dataset does not match any cluster in another dataset? Since you are using a match cutoff, if no cluster in the other dataset is matched how are the cells in this cluster corrected?

*Response: A very good point. If a cluster in one dataset does not match any clusters in any other datasets, we assume that this cluster is dataset-specific one, and the variation of the cells in this cluster is caused by both batch effects and biological difference. The correction vectors are learned from other shared clusters among different datasets. scMC then removes batch effects of the cells in this dataset-specific cluster by projecting the single-cell data onto these correction vectors. In the revised manuscript, we have clarified this point (Page 23).*

b. I was surprised that the correction terms are cluster specific. Why is that? Why not learn global variance correction terms? This way you can correct for all cells, whether they are in a matched cluster or not. This would also allow you to correct for additional data from the same lab when it becomes available rather than re-running the method from scratch.

*Response: Sorry for the confusion. The correction vectors are not cluster specific, and they are learned by subtracting the inferred technical variation from the global variance. The technical variation is indeed inferred by identifying the shared cell clusters across datasets, which is crucial for allowing to explicitly discriminate technical variation from biological variance in scMC. Although the correction vectors are learned from confident cells in scMC, they are used for correcting all cells, as shown in the subsection “Construction of corrected data for downstream analysis” in the Methods section. This point has also been emphasized in the section of “Overview of scMC” in the Results section. In the revised manuscript, we also have clarified this point in the Methods section (page 23).*

*The learned correction vectors can also be directly used for correcting the batch effects when additional data becoming available. In the revised manuscript, we have tested scMC on the simulation dataset 2 with three batches. We run scMC on the cells from the first two batches, and then project the cells from the third batch onto the shared UMAP space learned for the first two batches using the learned correction vectors. As expected, the cells with the same labels from the third batch are correctly placed onto this shared UMAP space (new Additional File 2 Figure S16). However, projecting cells from very different sources with strong batch effects into a shared low-dimensional space may require a more advanced transfer learning approach. We have clarified this point in the Discussion section (page 17).*

c. How many genes were selected as features for each of the datasets you analyzed? Did you try other thresholds for selecting such features? Since correction is only learned for selected genes, if they are not included there will be no way to study them in the joint space.

*Response: A very good point. The number of highly variable genes (HVGs) that are selected as features is typically around 2000-3000 in our study. The thresholds we chose are the typical ones that are widely used in other single cell analysis tools such as Seurat (Stuart et al, Cell 2019). To further analyze the effect of the thresholds, in the revised manuscript we tested two more thresholds for HVGs selection when analyzing simulation dataset 1 (leading to 4457 and 10000 genes) and the mouse skin wound healing dataset (leading to 4792 and 17912 genes). Consistent results have been found among the three thresholds for HVGs selection (Additional File 2 Figure S19).*

*We agree with the reviewer that correcting more or all genes is needed in order to study details of those genes in the joint space, for example, visualization of expression profile of individual gene in the integrated UMAP space. However, using highly variable genes instead of all genes can improve the performance of analysis because of the noisy characteristics of single cell data and computational cost. The correction learned from selected high variable genes is often sufficient to project all cells in the shared space. A recent large-scale benchmarking study also showed that highly variable genes selection improves the performance of data integration compared to the full gene set (Luecken et al, bioRxiv 2020.05.22.111161). We have added a discussion on this point in Discussion section (Page 17).*

d. Can you explain the following sentence 'The number of putative cell clusters is determined by the number of singular values associated with the consensus matrix at which the increase ratio between adjacent singular values is smaller than 0.01.'? How many clusters do you have for each of the datasets? Are some cell types divided between multiple clusters or do you usually see a single cluster for each type?

*Response: Sorry for the confusion. To determine the number of cell clusters, we build a consensus matrix by varying the resolution parameter values in the Leiden clustering algorithm. Each entry in the consensus matrix varies from 0 to 1 and represents the probability of two cells being in the same cluster. The number of cell clusters can be approximated by the number of larger singular values that capture the larger variance of the consensus matrix via singular value decomposition. Specifically, we calculate the ratio of each singular value to the sum of all the singular values, which indicates the percentage of variance each singular value contributes to. The number of singular values that have the ratio higher than 1% is used to estimate the number of putative cell clusters.*

*In the studied datasets, the number of clusters ranges from 3 to 20. We usually see a single cluster for each cell type; however, using this unsupervised method, some cell types might be divided into multiple clusters due to the inherit heterogeneity and the existence of different cellular states.*

*We have clarified those details in the Methods section (Page 20).*

e. Selection of marker genes for cluster matching: Is this done only on the confident cells or using all cells in the cluster? If the latter, why? Since only the confident ones are used to learn the correction why not use them to determine the matching as well?

*Response: Sorry for the confusion. We indeed performed clustering matching on the confident cells. This information was indicated in the Results section in the original manuscript. We have made this point clearer in the Methods section in the revised manuscript (Page 21).*

f. Selection of marker genes: What likelihood ratio tests are you referring to here? One vs. all? What exactly is being compared to identify these markers?

*Response: We used a likelihood ratio test that was proposed in a previous study for single-cell gene expression analysis (McDavid et al., Bioinformatics, 2013). Marker genes are identified by comparing the gene expression of confident cells in a particular cell cluster with all other cell clusters. This method can simultaneously test for changes in mean expression (conditional on the gene being expressed) and in the percentage of expressed cells. In the revised manuscript, we have clarified this point (Page 21).*

g. Matching clusters: Can you have many to many matchings? If so, how do you handle the multiple correction factors you learn for the same class?

*Response: Yes, many-many matching might happen and scMC can deal with such case. In scMC, a set of global correction factors is learned by minimizing the sum of the technical variation calculated from all pairs of shared clusters, which is summarized in E.q. (1). The corrected data L is obtained by projecting the stacked data matrix with all cells onto the learned correction vectors V, i.e., L = XV, where each column of V represents a correction vector. In the revised manuscript, we have made this point clearer (Page 22 and Page 23).*

h. In the section: 'Learning the correction vectors', line 50 on page 16. I don't understand the new optimization function for V. Isn't it fixed already by the assignment from the above optimization function (top of page 16)?

*Response: Sorry for the confusion. These two optimization functions are the same but in different representations. We intended to represent the objective function in terms of a matrix form for better representing the single cell data input matrix and confounding matrix. We have re-phased this point to make it clearer in the revised manuscript (Page 22).*

i. (side comment - would have been very useful to number of the equations). Line 20 on page 16. This seems to be sensitive to the ordering of the cells, no? Why is this correct? Why not have a function that is independent of the ordering?

*Response: A very good point. In the revised manuscript, we have added number to each equation. The performance of scMC is generally not sensitive to the ordering of the cells. Since the expression profiles of the confident cells in each pair of clusters are similar, changing the cells ordering in X ̂_i^p and X ̂_j^q will only lead to a random noise perturbation of X ̂_i^p- X ̂_j^q. Of note, the optimization problem in Eq. (1) is the classic principal component analysis (PCA). Since we are interested in the inferred vectors with larger variance and PCA is known as an effective tool for random noise attenuation (Ma et al, Wireless Personal Communications 2018), such random noise perturbation likely has little influence on the inferred vectors. In our all tested examples, we did not find influence on the performance of scMC. We have clarified this point in the revised manuscript (Page 22).*

j. What do you mean by 'randomly subset' (line 11 on that page)? Do you mean that you remove a subset of the cells? Why? How is this affecting the results? Can you better explain that equation?

*Response: Sorry for the confusion. To ensure the minus operator is defined for the two submatrices that might have different number of cells (rows), we need to match the dimensions of the two submatrices. One possible way to match is to randomly sample the cells in the larger submatrix to form a smaller submatrix. Since the two original submatrices represent the confident cells of very similar genomic profiles in a pair of shared clusters, such random sampling has little influence on the final result. We found very little effect of such random sampling when performing different runs on the datasets we used in the manuscript. In the revised manuscript, we clarified this point (Page 22).*

k. LISI calculation - Why are you presenting the average LISI and not the two components separately? Basically cLISI is the same as specificity, why do you need both? Replace the LISI result with b and c LISI and remove specificity since its redundant with cLISI.

*Response: A good integration method should be able to remove batch effects as well as preserve biological differences among cell types. To assess both batch effect removal and cell type separation that are assessed by bLISI and cLISI, we summarized these two metrics into a single F1 score using a similar formular in a previous study (Lin et al, PNAS 2019). LISI-derived F1 score measures the trade-off between batch effect removal and cell type separation. In addition, in the revised manuscript we added analysis using additional 9 evaluation metrics that were used in a recent benchmarking paper (Luecken et al, bioRxiv 2020.05.22.111161). To present these multiple evaluation metrics in a consistent way, we retained the derived F1-scores in the figures, and presented the two separate metrics bLISI and cLISI in Additional file 2 Figures S15 and S16.*

*The Specificity metric is different from cLISI. The Specificity metric evaluates the level of preserving dataset-specific cell types, whereas cLISI evaluates the preservation of all cell types. If there are dataset-specific cell types across different datasets, both bLISI and cLISI are unable to evaluate whether the integration method can preserve such specific cell types. In the revised manuscript we have clarified the differences between different evaluation metrics in the Methods section (Page 27).*

2. The paper is very light on any biological insights that the new alignment provides. While this is a methods paper it would be good to discuss this a bit more, especially for the ATAC-Seq data.

*Response: Thanks for the nice suggestion. To demonstrate the biological insights that can be gained by scMC, in the revision we investigated 1) the functional differences between the new identified Hedgehog -active and the classic fibroblast subpopulations during mouse skin wound healing, 2) the pseudotemporal dynamics of epidermal and dermal differentiation during mouse embryonic development, and 3) the enriched biological processes and motifs for the differential loci of the identified subpopulations in the scATAC-seq dataset.*

*1) For the new identified Hedgehog -active fibroblast subpopulations during mouse skin wound healing, we show that the marker genes of these fibroblasts are enriched in vast different biological processes compared to the classic fibroblasts (i.e., the identified Hedgehog -inactive fibroblasts), which are typically involved in processes such as extracellular matrix (ECM) and collagen formation to support the effective wound healing. Nevertheless, Hedgehog -active fibroblasts are involved in processes such as ribosome biogenesis/assembly and rRNA processing (updated Figure 4e), implying active RNA dynamics and cell fate transition. Indeed, Hedgehog -active fibroblasts also express dermal papilla signature genes such as Igfbp4 and Wif1 (updated Figure 4d), suggesting that these fibroblasts are likely undergoing transition to dermal papilla, which is in agreement with the fact that dermal fibroblasts could give rise to dermal papilla cells during hair follicle formation (Driskell et al., Nature 2013, and Lim et al., Nature Communications 2018). Together, these results suggest the potential role of Hedgehog activation in inducing fibroblasts into dermal papilla cells. In the revised manuscript we added these results in the Results section (Page 10).*

*2) For the pseudotemporal trajectory analysis during mouse embryonic development, we show that scMC reveals the known differentiation trajectory, and recapitulates the known temporal dynamics of both upregulated and downregulated marker genes (updated Figure 5). In the revised manuscript we added these results in the Results section (Page 12).*

*3) For the scATAC-seq dataset, scMC identified four cell subpopulations in the brain tissue. We then aggregated chromatin accessible signals across each cell subpopulation and identified differential loci of these four cell subpopulations. By performing gene ontology enrichment analysis using GREAT and motif enrichment analysis by chromVAR, we found different enriched biological processes and motif patterns across these four different cell subpopulations (new Additional file 2 Figure S17). In the revised manuscript we added these results in the Results section (Page 14).*

3. Can you explicitly mention the run time for different sized (not just point to the supplement) and also mention if the run time is linearly or quadratically or any other complexity term related to the size of the input? Would the method work on 100K cells? 1M cells?

*Response: In the revision we have moved the run time analysis to the Results section (Page 7) and now clearly indicated the specific run time. The most time-consuming procedure of scMC is solving the optimization problem (Eq. (3)), which is equivalent to calculating k largest eigenvectors (default k=40) associated with the real positive eigenvalues of a n*n matrix, where n is the number of all confident cells. Therefore, the computational complexity is O(kn^2). To reduce the run time, users can use fewer confident cells by adjusting the quantile cutoff parameter (e.g. from 0.75 to 0.9) or perform subsampling of single-cell datasets using a ‘geometric sketching’ approach (Hie et al., Cell Systems 2019), which allows to maintain the transcriptomic heterogeneity within a dataset with a smaller subset of cells.*

*To investigate if scMC can handle large-scale datasets, we have tested scMC on an extended simulation dataset 1 with 100K cells, and the run time is 1.4 hour. However, we did not test it on the dataset with 1M cells because we failed to simulate such dataset using Splatter R package on our computer, which was equipped with Intel Xeon E5-2690 v.3 processors and 64GB of memory. In the revision we have added the computational complexity and the analysis of a large-scale dataset in the Discussion section (Page 16).*

4. There are several typos in the paper and it would greatly benefit from English editing. A few examples:

' Due to rapid technological development, the single cell datasets have been generated under different' -> 'Due to rapid technological development, single cell datasets have been generated under different'

'both Seurat V3 and scMC well removed batch effects' -> 'both Seurat V3 and scMC successfully removed batch effects' (several places)

'We then respectively reconstructed the epidermal and dermal trajectories' -> 'We then reconstructed the epidermal and dermal trajectories respectively'

*Response: Thanks for the careful reading. We apologize for not being more careful in proofreading and language polishing. In the revision we have corrected these typos and made significant effort in these two areas.*

**Second round of review**

**Reviewer 2**

The authors successfully addressed all my technical comments and the methods and analyses they performed are much clearer now. The only issue that still remains is the biological validation. I mentioned this as a minor issue in my initial review since this is a methods paper. The authors added some more validation / analysis examples but I still do not see how these show that the alignment they are suggesting leads to new insights. Specifically, most of the biological analysis they show (including the sub-typing and the analysis of atac-seq motifs) can be done on the two (or more) data separately. Does the alignment lead to better / more significant results for any of these datasets when compared to running the same type of analysis separately for each one? This would be good to discuss.

**Authors Response**

**Point-by-point responses to the reviewers’ comments:**

Reviewer reports

Reviewer #2: The authors successfully addressed all my technical comments and the methods and analyses they performed are much clearer now. The only issue that still remains is the biological validation. I mentioned this as a minor issue in my initial review since this is a methods paper. The authors added some more validation / analysis examples but I still do not see how these show that the alignment they are suggesting leads to new insights. Specifically, most of the biological analysis they show (including the sub-typing and the analysis of atac-seq motifs) can be done on the two (or more) data separately. Does the alignment lead to better / more significant results for any of these datasets when compared to running the same type of analysis separately for each one? This would be good to discuss.

*Response: We thank the reviewers’ effort and constructive comments for improving our manuscript. We agree that many of the results obtained by joint analysis might be obtained by individual analysis of each sample with additional careful effort in comparing them. However, for some cases our alignment method could provide biological insights that are difficult to obtain if only analyzing datasets individually. For example, for the time-course embryonic skin development dataset, by reconstructing the integrated trajectory using both E13.5 and E14.5 epidermal and dermal cells, we found a full range of epidermal and dermal differentiation programs are established as early as E13.5, and consistently, the E14.5 cells are exclusively made up the terminus of the pseudotime trajectory. Without aligning the two datasets on one integrated trajectory, it would be difficult to obtain this important biological insight. For the mouse skin wound system, while the novel Hedgehog-active fibroblast subpopulation could be uncovered by analyzing the datasets individually, the question on whether this subpopulation is a specific fibroblast subpopulation upon Hedgehog activation would be difficult to answer without using our alignment approach. In general, the apparent differences seen between two individual analysis sometimes might be caused by technical variation. The capability of distinguishing technical variation from biological variation in scMC is critical when comparing cellular compositions and trajectories across conditions and time points, which are often measured in different batches with potential technical variation.*

*In this revision we have added a paragraph on this point in Discussion on Page 16.*
